# Costs of Delivering the Hep B PAST Model of Care in Remote Australia: A Retrospective Pre–Post Study

**DOI:** 10.5694/mja2.70292

**Published:** 2026-09-30

**Authors:** Hoa Nguyen, Anh Le Tuan Nguyen, Paula Binks, Melita McKinnon, Emily Vintour‐Cesar, Karen Wills, Nicola Stephens, Benjamin Cowie, Joshua S. Davis, Yuejen Zhao, Peter Nihill, Julie A. Campbell, Andrew J. Palmer, Jane Davies, Barbara de Graaff

**Affiliations:** ^1^ Menzies Institute for Medical Research University of Tasmania Hobart Tasmania Australia; ^2^ WHO Collaborating Centre for Viral Hepatitis, The Peter Doherty Institute for Infection and Immunity Melbourne Victoria Australia; ^3^ Department of Health Economics Hanoi Medical University Vietnam; ^4^ Global and Tropical Health Division Menzies School of Health Research Tiwi Northern Territory Australia; ^5^ School of Medicine University of Tasmania Hobart Tasmania Australia; ^6^ Victorian Infectious Diseases Service Royal Melbourne Hospital Parkville Victoria Australia; ^7^ Northern Territory Department of Health Casuarina Northern Territory Australia; ^8^ School of Medicine and Public Health University of Newcastle Newcastle New South Wales Australia

**Keywords:** cost–benefit analysis, delivery of healthcare, hepatitis B, indigenous health

## Abstract

**Objectives:**

To estimate the direct medical costs of the Hep B Partnership Program (Hep B PAST) model of care compared with chronic hepatitis B (CHB) usual care from the health system perspective (Northern Territory (NT) Department of Health and the Commonwealth).

**Study Type:**

Retrospective cohort study (2014–2023) that linked primary care with inpatient, emergency, outpatient and outreach datasets. Costs were applied using National Weighted Activity Units, the Pharmaceutical Benefits Scheme and NT Government costings. A pre‐ and post‐implementation design from a health system perspective was applied, with costs in 2023 Australian dollars.

**Setting:**

Remote communities in the NT, Australia.

**Participants:**

The cohort included all First Nations people living with CHB who were attending a clinic in a remote community that had consented to the Hep B PAST program, and who received care between 1 July 2014 and 31 May 2023.

**Main Outcome Measures:**

Annual mean and median healthcare costs per contact and per individual across service types, with comparisons of annual mean costs per individual between pre‐ and post‐Hep B PAST using partially overlapping *t*‐tests.

**Results:**

Among 1063 First Nations individuals with CHB, annual median costs per individual were similar for usual care ($639 [interquartile range, $366–1156]) and Hep B PAST ($770 [interquartile range, $400–1272]); mean costs were also similar for usual care versus Hep B PAST ($1701 vs. $1970; *p* = 0.12). By service type, annual median costs were similar for inpatient services ($7777 vs. $7795), primary care ($327 vs. $302) and outpatient visits ($1985 vs. $1858), and higher for Hep B PAST for emergency services ($1339 vs. $1672) and outreach care ($373 vs. $746).

**Conclusions:**

In remote First Nations communities, Hep B PAST was delivered with a modest increase in direct medical costs compared with usual care. These findings can be used to inform future cost‐effective evaluations. Future cost savings from decreased hepatocellular carcinoma and liver failure were not included.

## Plain Language Summary

**The Known:**

Chronic hepatitis B (CHB) disproportionately affects Australia's First Nations peoples, particularly in the Northern Territory. The Hep B PAST program has strengthened diagnosis, monitoring and treatment uptake through a culturally responsive approach.

**The New:**

Hep B PAST delivers high‐quality CHB care in remote communities with a modest increase in direct healthcare costs compared with usual care.

**The Implications:**

This study provided economic evidence supporting Hep B PAST as a model that can be scaled to improve CHB care in remote First Nations communities. Policy‐makers can use this evidence to justify investment in decentralised service delivery to advance health equity and CHB elimination targets.

## 1 Introduction

Chronic hepatitis B (CHB) remains a major cause of preventable liver‐related morbidity and mortality worldwide, primarily through hepatocellular carcinoma and cirrhosis [1]. In Australia, an estimated 219,800 people were living with CHB in 2023, representing 0.8% of the total population [2, 3]. The burden of disease is unevenly distributed, with the Northern Territory (NT) reporting the highest prevalence nationally (1.8%) [3], where about three‐quarters of those affected are First Nations peoples [2, 4].

In 2018, the Hep B Partnership Program (Hep B PAST) was established in response to First Nation communities' concerns regarding the impact of liver disease. The program included improved CHB health literacy via the Hep B Story app [5, 6], strengthened care coordination via the Hep B Hub facilitation tool [7] and extensive training for healthcare providers [8, 9]. A specialist outreach service was also implemented, where a team of infectious diseases physicians, sonographers and clinical nurse specialists provided care within communities [6, 7]. This means an individual can have their ultrasound, FibroScan, clinical review, blood tests and education all in one visit and this care is delivered ‘on country’ close to their home. Before Hep B PAST, an individual would have separate visits for each component of care and would need to travel on average 1–2 h on a small plane to access ultrasound and FibroScan. Between 2018 and 2023, Hep B PAST achieved high uptake of CHB care, with nearly 100% of participants aware of their CHB status, 86.3% engaged in care and 24.1% receiving antiviral treatment in 2023 [6]. The Fourth National Hepatitis B Strategy has acknowledged this model of care and recommended this approach for expansion and national scale up [10].

With ongoing increases in healthcare costs, health economic evaluations are vital for informing resource allocation [11]. Such evidence can be particularly valuable for groups who experience health inequities, such as Australia's First Nations peoples, to indicate where resources are best placed to help bridge health inequities [11, 12]. Therefore, this study aimed to estimate the direct medical costs of Hep B PAST, and compare these with the costs of CHB usual care, from the health system perspective.

## 2 Methods

A retrospective cohort study using linked, longitudinal health and administrative data was conducted. All cost estimates were adjusted to the financial year 2023–2024 using Consumer Price Index inflators [13] and expressed in 2023 Australian dollars. This study is reported in accordance with the CHEERS 2022 reporting guidelines (Table S1).

### 2.1 Setting and Cohort

The NT covers a large land mass with a small and dispersed population of 232,605 people in 2021 [14]. First Nations peoples represent 26.3% of the NT population, most living in remote and very remote areas [12, 14]. The Hep B PAST program was piloted in 2016 and gradually implemented across 66 of the 87 remote community clinics in the NT between 2018 and 2023.

The study cohort included all First Nations people living with CHB who were attending a clinic in a remote community that had consented to the Hep B PAST, and who received care between 1 July 2014 and 31 May 2023. The process for determining the final cohort is shown in Figure 1, and further details are provided in the Supporting Information. Sex was defined as biological sex (male or female) as recorded in clinical and administrative datasets and is used consistently throughout.

**FIGURE 1:**
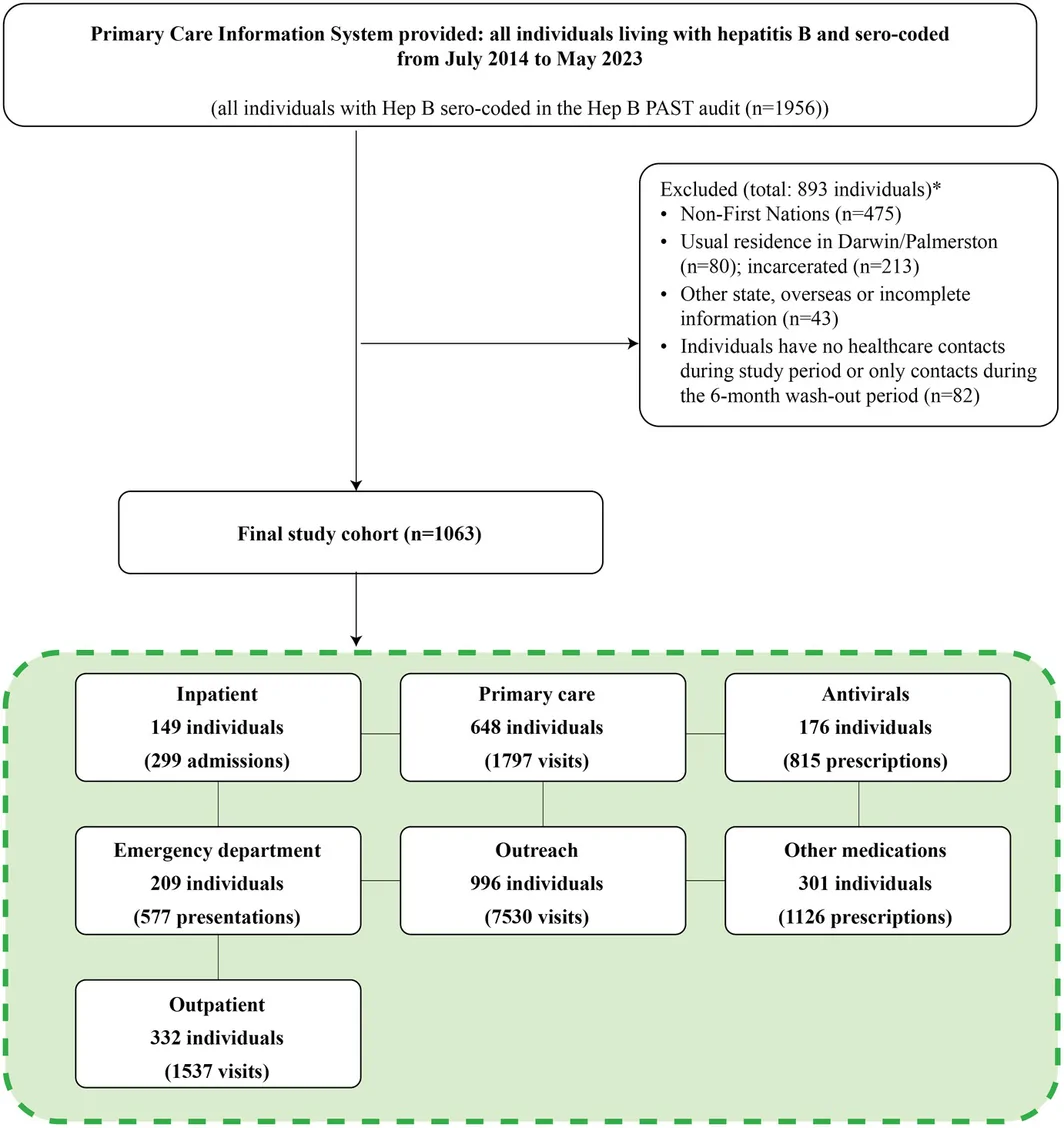
Selection of the study cohort. Hep B PAST, Hep B Partnership Program. ^*^Exclusion criteria are not mutually exclusive, individuals may have one or more reasons for exclusion.

### 2.2 Costing Methods

This study was performed from a health system perspective (NT and the Commonwealth), reflecting the payers of these costs. For this study, ‘healthcare contact’ refers to any interaction an individual has with the healthcare system.

#### 2.2.1 Hospital Services

Hospital services were costed using National Efficient Prices (NEPs) [15] and National Weighted Activity Units (NWAUs) published by the Independent Health and Aged Care Pricing Authority [16]. Inpatient services were classified using Australian Refined Diagnosis Related Groups [17]; emergency department (ED) services using Urgency Related Groups (2014–2020) [18] and Urgency Disposition Groups (2021–2023) [18]; and outpatient services using the Tier 2 Non‐Admitted Services Classification [19]. Costs were estimated by multiplying the NWAU allocated to the appropriate service by the NEP for that financial year. Interstate hospital admissions were also identified and costed.

Only healthcare contacts mentioning hepatitis B or its sequelae (e.g., hepatocellular carcinoma, cirrhosis, ascites) were included, with diagnostic codes listed (Table S2).

Hep B PAST delivered specialist care through outpatient services, including telehealth and in‐person visits requiring travel to urban centres. Travel costs subsidised through the Patient Assistance Travel Scheme were estimated at $1 per kilometre, consistent with previous research [20, 21].

#### 2.2.2 Outreach Services

The Hep B PAST outreach service delivers care to remote communities via scheduled visits from the capital of the NT, Darwin [7]. Outreach costs were estimated from program budgeting documents, assuming 15 patients per visit and a per‐patient cost of $450.

#### 2.2.3 Primary Care Services

CHB care in remote primary healthcare clinics is provided by Aboriginal health practitioners, nursing staff and medical personnel, with support provided through visits and telehealth, facilitated by the Hep B Hub. Costs of primary care per financial year were obtained from an internal NT Department of Health report (unpublished). The mean cost per visit was $266 in 2017–2018, $256 in 2019–2020 and $226 in 2021–2022.

#### 2.2.4 Antiviral and Other Liver‐Related Medications

Information on CHB antivirals and other prescribed medications related to liver diseases were extracted from the Primary Care Information System database. A list of all prescribed medications, excluding antivirals, was independently reviewed by two clinicians (JSD, JD) to determine whether they were likely prescribed for sequelae of hepatitis B. Antiviral medications were analysed separately as substantial reductions in unit costs over the study period. Medication costs were extracted from Pharmaceutical Benefits Scheme (PBS) data based on the prescription date [22]. Unit costs and assumptions are provided in Table S3.

### 2.3 Pre‐ and Post‐Design

A pre‐ and post‐implementation design with a 6‐month wash‐out period was adopted to reduce potential contamination (e.g., increased service provision before implementation due to awareness of the planned program). The pre‐Hep B PAST costs were calculated from July 2014 to 6 months before implementation at each clinic. The post‐Hep B PAST costs were calculated from 6 months after the implementation date to May 2023.

### 2.4 Sensitivity Analysis

As costs for outreach and primary care were estimated from the program budgeting documents and the internal NT Department of Health report, sensitivity analyses were undertaken to explore the impact of varying these costs. Outreach costs were varied from $400 and $500 per visit around the baseline of $450. For primary care, costs were available for financial years 2017–2018, 2019–2020 and 2021–2022, with costs for other periods extrapolated assuming annual changes of ±$5.

### 2.5 Statistical Analysis

Descriptive statistics are used to summarise the demographic data. Mean values with 95% confidence intervals (CIs) and median values with interquartile ranges (IQRs) are reported for continuous variables, and frequencies with percentages are reported for categorical data. Differences in annual mean costs per individual between pre‐ and post‐Hep B PAST were assessed using partially overlapping *t*‐tests implemented in R (v4.2.2) [23] with the *Partiallyoverlapping* package [24, 25].

### 2.6 Ethics Statement

Ethical approvals were provided by the Human Research Ethics Committee of the NT Department of Health and Menzies School of Health Research (NT HREC 2018–3242) and the University of Tasmania Human Research Ethics Committee (28696).

## 3 Results

The study cohort included 1063 First Nations people with CHB, of whom 56% (596/1063) were male. Median age increased from 46 years (IQR, 36–56 years) (calculated at January 2018) to 51 years (IQR, 42–62 years) by the end of the study (May 2023). Based on clinic participation and timing of participation, 859 individuals were included in the pre‐Hep B PAST group and 931 individuals in the post‐Hep B PAST group (Table 1). The age group distributions are provided in Table S4.

**TABLE 1:** Demographic overview of the study cohort pre‐ and post‐Hep B PAST.

Financial year	Pre‐Hep B PAST			Post‐Hep B PAST		
	Total, *n*	Male, *n* (%)	Female, *n* (%)	Total, *n*	Male, *n* (%)	Female, *n* (%)
2014–2015	437	232 (53%)	205 (47%)	0	0	0
2015–2016	522	284 (54%)	239 (46%)	0	0	0
2016–2017^a^	459	251 (55%)	208 (45%)	37	19 (51%)	18 (49%)
2017–2018	408	226 (55%)	182 (45%)	107	62 (58%)	45 (42%)
2018–2019	188	112 (60%)	76 (40%)	295	155 (53%)	140 (47%)
2019–2020	110	64 (58%)	46 (42%)	571	314 (55%)	258 (45%)
2020–2021	0	0	0	640	350 (55%)	290 (45%)
2021–2022	0	0	0	679	374 (55%)	305 (45%)
2022–2023	0	0	0	601	339 (56%)	262 (44%)
Total	859	479 (56%)	380 (44%)	931	522 (56%)	409 (44%)

Abbreviation: Hep B PAST, Hep B Partnership Program.

^a^ Data for 2016–2017 include pilot implementation in five remote communities.

### 3.1 Inpatient Services

In the pre‐Hep B PAST group, 71 individuals had 123 liver‐related admissions, with a median cost per admission of $6758 (IQR, $2159–12,236) and an annual median cost per individual of $7777 (IQR, $4555–18,274). In the post‐Hep B PAST group, 91 individuals had 176 admissions and the median cost per admission decreased to $5376 (IQR, $2401–11,724), while the annual median cost per individual remained similar at $7795 (IQR, $2120–19,813) (Figure 2a). The overlapping *t*‐test of mean costs provided similar mean costs per individual per year: $13,776 (95% CI, $10,461–17,091) for pre‐Hep B PAST and $15,745 (95% CI, $11,964–19,527) for post‐Hep B PAST (*p* = 0.65, Table 2).

**FIGURE 2:**
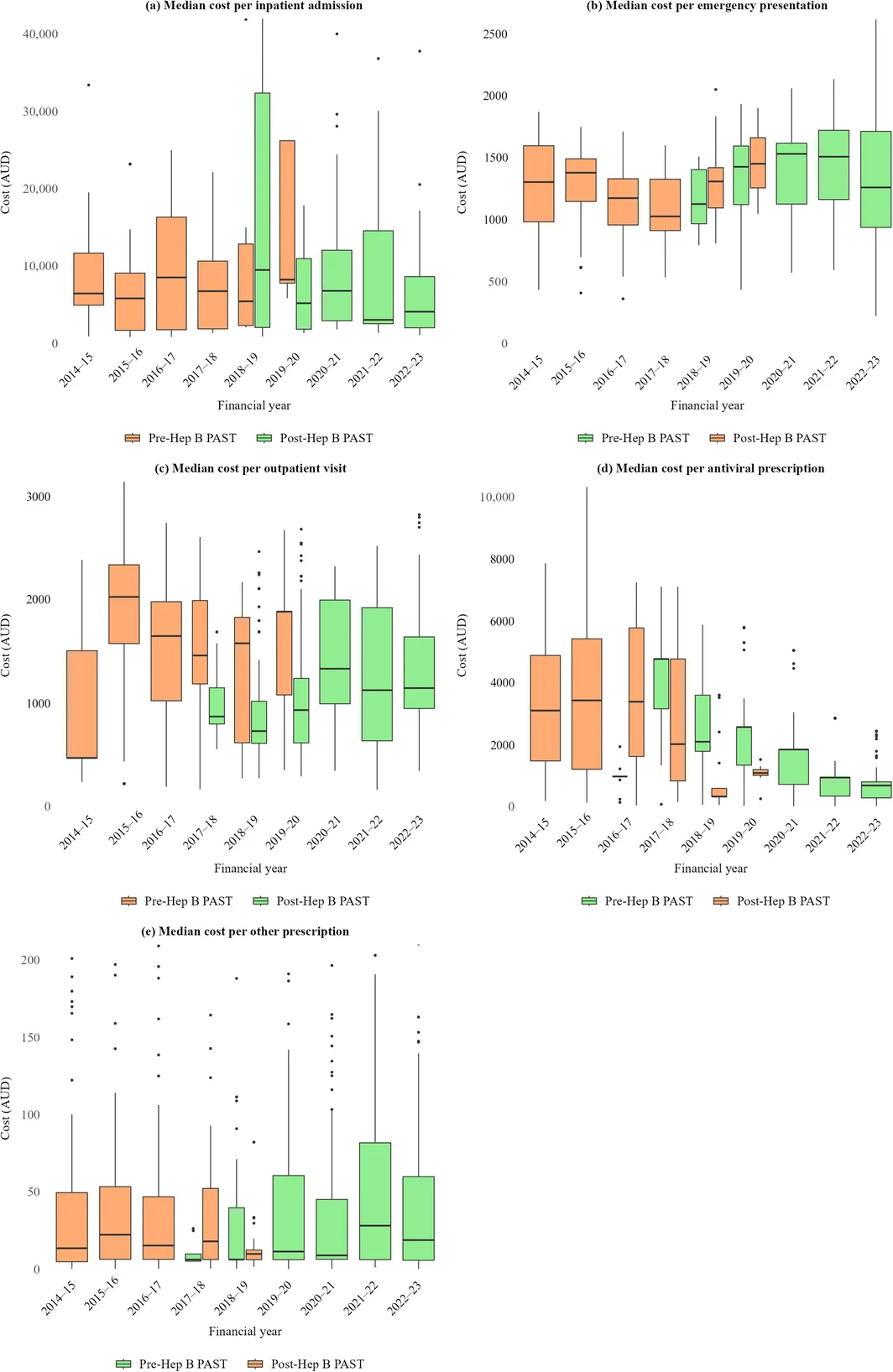
Median cost per contact by financial year across different healthcare services, pre‐ and post‐Hep B PAST.^a^ AUD, Australian dollars; Hep B PAST, Hep B Partnership Program. ^a^Boxes represent the interquartile range (25th–75th percentiles), with horizontal lines indicating median values. Whiskers indicate the range of non‐outlier observations, and dots indicate outliers. Green and orange boxes represent the pre‐ and post‐Hep B PAST, respectively.

**TABLE 2:** Comparison of annual mean cost per individual pre‐ and post‐Hep B PAST by service type.

Service type	Mean (95% CI) cost in Australian dollars		*p* ^a^
	Pre‐Hep B PAST	Post‐Hep B PAST	
Inpatient services	$13,776 ($10,461–$17,091)	$15,745 ($11,964–$19,527)	0.65
Emergency department services	$1685 ($1487–$1882)	$2679 ($2223–$3136)	0.002
Primary care services	$425 ($400–$450)	$392 ($377–$407)	< 0.001
Outpatient services	$2972 ($2230–$3713)	$4999 ($3606–$6391)	0.02
Outreach services	$581 ($566–$597)	$751 ($732–$770)	< 0.001
Antiviral medications	$3283 ($2912–$3655)	$1639 ($1525–$1753)	< 0.001
Other medications	$57 ($49–$66)	$163 ($92–$234)	0.11
All services (excluding antivirals)	$1701 ($1459–$1942)	$1970 ($1721–$2218)	0.12

Abbreviations: CI, confidence interval; Hep B PAST, Hep B Partnership Program.

^a^ *p* values derived from partially overlapping *t*‐tests.

### 3.2 Emergency Department Services

In the pre‐Hep B PAST group, 126 individuals had 253 ED presentations, with a median cost per episode of $1222 (IQR, $1002–1490) and an annual median cost per individual of $1339 (IQR, $1032–1818). In the post‐Hep B PAST group, 125 individuals had 324 ED presentations; the median cost per presentation was $1467 (IQR, $1090–1682) and the annual median cost per individual was higher at $1672 (IQR, $1246–2668) (Figure 2b). Comparing mean costs showed higher annual mean costs per individual for ED services for post‐Hep B PAST: $2679 (95% CI, $2223–3136) compared with $1685 (95% CI, $1487–1882) for pre‐Hep B PAST (*p* = 0.002, Table 2).

### 3.3 Outpatient Services

In the pre‐Hep B PAST group, 222 individuals had 549 outpatient visits, with a median cost per visit of $1659 (IQR, $1005–2032) and an annual median cost per individual of $1985 (IQR, $1458–2054). In the post‐Hep B PAST group, 149 individuals attended 988 outpatient visits, the median cost per visit decreased to $1089 (IQR, $750–1859) and the annual median cost per individual decreased to $1858 (IQR, $1045–3584) (Figure 2c). In contrast, higher annual mean costs per individual for outpatient services were observed for post‐Hep B PAST ($4999 [95% CI, $3606–6391]) compared with pre‐Hep B PAST ($2972 [95% CI, $2230–3713]) (*p* = 0.02, Table 2).

Following Hep B PAST, telehealth use increased from 11.1% (61/549) to 30.6% (302/988) of outpatient contacts, while face‐to‐face visits decreased proportionally despite higher absolute numbers, from 88.9% (488/549) to 69.4% (686/988). Travel costs also increased, from $611,226 to $748,951.

### 3.4 Outreach Services

In the pre‐Hep B PAST group, 770 individuals accessed outreach care (2767 visits), with a median annual cost per person of $373 (IQR, $362–732). In the post‐Hep B PAST group, this increased to 856 individuals and 4763 visits, with a higher median cost per person of $746 (IQR, $386–848). Since a single cost of providing an outreach visit was used, median and IQR values per visit are not appropriate. Mean annual costs per individual were higher for post‐Hep B PAST ($751 [95% CI, 732–770]) than for pre‐Hep B PAST ($581 [95%, CI, 566–597]) (*p* < 0.001, Table 2).

### 3.5 Primary Care Services

In the pre‐Hep B PAST group, 287 individuals attended primary care services, accounting for 476 visits. The annual median cost per individual was $327 (IQR, $325–327). In the post‐Hep B PAST group, the number of individuals increased substantially to 516, with 1321 visits. The median cost per individual was marginally lower at $302 (IQR, $257–492). As a single cost was used, median and IQR values per visit are not appropriate. Mean annual costs per individual were lower for post‐Hep B PAST ($392 [95% CI, $377–407]) than for pre‐Hep B PAST ($425 [95% CI, $400–450]) (*p* < 0.001, Table 2). Counts, contacts and cost summaries are provided (Table S5).

### 3.6 Antiviral Treatments

In the pre‐Hep B PAST group, 61 individuals had 187 prescriptions, with a median annual cost per individual of $2766 (IQR, $1204–5342). In the post‐Hep B PAST group, 161 individuals had 628 prescriptions with lower cost of $964 (IQR, $741–2426), reflecting PBS drug price reductions (Figure 2d; Table S6). Lower annual mean costs per individual were observed for antiviral medications for post‐Hep B PAST ($1639 [95% CI, $1525–1753]) compared with pre‐Hep B PAST ($3283 [95% CI, $2912–3655]) (*p* < 0.001, Table 2).

### 3.7 Other Liver Disease‐Related Medications

In the pre‐Hep B PAST group, 162 individuals had 450 prescriptions, with a median annual cost per individual of $26 (IQR, $6–80). In the post‐Hep B PAST group, 216 individuals had 676 prescriptions, with a median cost per individual of $24 (IQR, $6–96) (Figure 2e; Table S6). The mean annual cost of other liver disease‐related medications increased from $57 (95% CI, $49–66) to $163 (95% CI, $92–234) per person; however, this difference was not statistically significant, likely due to high variability in the data (*p* = 0.11, Table 2).

### 3.8 Total Costs (Excluding Antivirals)

Antiviral medications were excluded from total cost estimates due to substantial price reductions over the study period. In the pre‐Hep B PAST group, 859 individuals had 4618 healthcare contacts, with an estimated total cost of $3,581,264. The median costs were $366 per contact (IQR, $356–380) and $639 per individual (IQR, $366–1156). In the post‐Hep B PAST group, 926 individuals had 8248 contacts, resulting in a total cost of $5,680,946. The median costs per contact and per individual were $386 (IQR, $366–424) and $770 (IQR, $400–1272), respectively. Lastly, the partially overlapping *t*‐test of mean costs provided similar mean costs per individual per year: $1701 (95% CI, $1459–1942) for pre‐Hep B PAST and $1970 (95% CI, $1721–2218) for post‐Hep B PAST (*p* = 0.12, Table 2).

### 3.9 Sensitivity Analyses

Varying outreach and primary care costs changed absolute total costs but did not alter overall cost patterns. With outreach costs ranging from $400 to $500 per visit, total non‐antiviral costs were $3.5–3.7 million pre‐Hep B PAST and $5.5–5.9 million post‐Hep B PAST, with mean annual costs per person of $1647–$1754 in the pre‐Hep B PAST group and $1897–$2043 in the post‐Hep B PAST group.

Varying primary care costs (±$5 annually) resulted in total costs of $3.6 million pre‐Hep B PAST and $5.7 million post‐Hep B PAST, with mean annual costs remaining stable ($1703 pre‐Hep B PAST and $1972 post‐Hep B PAST) (Table S7).

## 4 Discussion

This study has shown that novel, high‐quality healthcare can be provided in remote settings of Australia without incurring substantially higher costs. This is important for several reasons; most importantly, many people living in remote First Nations communities experience inequitable access to healthcare, which, in turn, contributes to poorer health outcomes [10, 26].

In Australia, previous model‐based studies have suggested that increased investment in CHB care is needed to meet elimination targets [27, 28, 29]. While our study aligns with this, the increased costs were surprisingly marginal: the annual mean cost per individual increased from $1701 to $1970 and no statistically significant difference was observed. Notably, individuals received more holistic and complete guideline‐based care following implementation of Hep B PAST, which has translated into improved clinical care uptake [6].

For primary care services, the number of individuals receiving liver‐related care increased from 287 to 516, with visits nearly tripling (from 476 to 1321). This suggests that the upskilling of the workforce has supported more CHB care to be undertaken in primary care. Over this period, the annual median and mean costs per individual were lower post‐Hep B PAST. Regarding outreach services, the annual mean cost per individual increased substantially from $581 pre‐Hep B PAST to $751 post‐Hep B PAST; however, the number of individuals accessing this type of service did not increase greatly (770 and 856, respectively). This reflects increased frequency and broader scope of care received by patients from the outreach team post‐Hep B PAST.

The cost of an inpatient admission is influenced by several factors, including admission complexity, length of stay and assigned Australian Refined Diagnosis Related Groups [17]. We observed that the median cost per inpatient admission declined from $6758 pre‐Hep B PAST to $5376 post‐Hep B PAST. We hypothesise that this may reflect increased community‐based care contributing to less complex hospital admissions. Further evaluation of the Hep B PAST program is required to better understand this.

For ED presentations, the annual mean cost per individual increased marginally ($1685 to $2679) while the number of patients remained stable (126 pre‐Hep B PAST and 125 post‐Hep B PAST). Because ED costs are primarily driven by the urgency and complexity of presentations [18], this increase may be partly explained by an ageing cohort with potentially greater clinical complexity and comorbidity burden in the post‐Hep B PAST group. Annual mean ED costs per individual were comparable to inflation‐adjusted estimates from a Victorian study ($2490) [30].

For outpatient services, the increase in annual mean cost per individual reflects a higher number of outpatient contacts per individual. This was accompanied by a substantial expansion of outpatient services via telehealth, improved access to specialised services for patients requiring long‐term management and greater patient engagement after Hep B PAST was implemented.

The number of individuals receiving antiviral therapy increased from 61 pre‐Hep B PAST to 161 post‐Hep B PAST, while the annual median antiviral cost per individual declined from $2766 to $964, largely reflecting progressive reductions in PBS prices rather than a single discrete price change. In Australia, CHB antivirals are PBS listed and government expenditure per prescription decreased substantially over the study period; for example, the price of entecavir 500 mg fell from $769 in 2014 [31] to $103 in 2023 [32]. These price reductions are critically important in supporting Australia's efforts to achieve elimination targets.

### 4.1 Strengths and Limitations

The study has several strengths, including linkage of de‐identified longitudinal datasets across multiple services and the use of NT‐specific primary care cost estimates, providing a more accurate reflection of healthcare costs in remote communities and enhancing the relevance of the findings for the NT context.

In terms of limitations of the study, outreach costs were estimated from program budgeting documents and may not capture visit‐level variability, although sensitivity analyses showed limited impact on overall costs. Also, inclusion of non‐antiviral medications with multiple indications may have resulted in some overestimation of CHB‐related medication costs. For example, furosemide may be used as a treatment for advanced liver disease and for heart failure. Finally, as the study did not use a random sample, but rather data from clinics participating in the Hep B PAST project, this may limit the generalisability of our findings to other settings.

## 5 Conclusions

The study demonstrates that the Hep B PAST model is economically feasible, delivering enhanced CHB care with a modest increase in direct healthcare costs. The findings from this study will inform future economic modelling to evaluate the cost‐effectiveness of the Hep B PAST model of care.

## Author Contributions

**Hoa Nguyen:** conceptualization, methodology, data curation, formal analysis, investigation, visualization, writing – original draft, writing – review and editing. **Anh Le Tuan Nguyen:** conceptualization, methodology, supervision, writing – review and editing. **Paula Binks:** conceptualization, clinical investigation, clinical interpretation, methodology, writing – review and editing. **Melita McKinnon:** data curation, validation, project administration, writing – review and editing. **Emily Vintour‐Cesar:** project administration, investigation, data curation, writing – review and editing. **Karen Wills:** methodology, validation, writing – review and editing. **Nicola Stephens:** conceptualization, supervision, writing – review and editing. **Benjamin Cowie:** conceptualization, methodology, clinical investigation, clinical interpretation, writing – review and editing. **Joshua S. Davis:** conceptualization, clinical investigation, clinical interpretation, writing – review and editing. **Yuejen Zhao:** methodology, data acquisition, interpretation of health data, writing – review and editing. **Peter Nihill:** resources, data acquisition, data interpretation, writing – review and editing. **Julie A. Campbell:** conceptualization, investigation, writing – review and editing. **Andrew J. Palmer:** conceptualization, investigation, writing – review and editing. **Jane Davies:** conceptualization, clinical investigation, clinical interpretation, methodology, resources, writing – review and editing. **Barbara de Graaff:** conceptualization, methodology, investigation, supervision, resources, writing – review and editing.

## Funding

Hep B PAST was supported by an Australian Government National Health and Medical Research Council partnership grant—GNT1151837. Funders played no role in the study design, data collection, analysis, interpretation, manuscript preparation or decision to publish.

## Conflicts of Interest

The authors declare no conflicts of interest.

## Supporting information


**Table S1:** CHEERS 2022 checklist.
**Table S2:** Principal and additional diagnosis codes (ICD‐10‐AM) for inpatient admissions and ED presentations included in the study.
**Table S3:** Unit costs and assumptions used in this study.
**Table S4:** Age group distribution of the study cohort pre‐and post‐Hep B PAST.
**Table S5:** Annual number of individuals, contacts and mean/median costs per individual and per contact for each healthcare service pre‐and post‐Hep B PAST.
**Table S6:** Summary costs of CHB antiviral and other liver‐related medications.
**Table S7:** Sensitivity analyses of total healthcare costs for outreach and primary care services pre‐and post‐Hep B PAST.

## Data Availability

The data are not publicly available because of the ethical, privacy and data custodian requirements governing individual‐level health data.

## References

[1] World Health Organization. Global hepatitis report , Action for Access in Low‐and Middle‐Income Countries (World Health Organization, 2024), accessed July 2026, https://www.who.int/publications/i/item/9789240091672.

[2] J. H. MacLachlan , A. Mondel , I. Purcell , and B. C. Cowie , Viral Hepatitis Mapping Project: Hepatitis B National Report 2023 (ASHM, 2025), accessed May 2026, https://ashm.org.au/wp‐content/uploads/2025/04/HBV_ViralHepReport_2023_Updated_14042025.pdf.

[3] A. Nguyen , N. Romero , J. H. MacLachlan , and B. C. Cowie , Surveillance for Hepatitis B Indicators: Tracking Australia's Progress Towards Hepatitis B Elimination – National Report 2023 (2025), accessed May 2025, https://www.doherty.edu.au/wp‐content/uploads/2026/01/National_Surveillance_for_Hepatitis_B_Indicators_2023_Final_with_cover‐1.pdf.

[4] A. Nguyen , N. Romero , J. H. MacLachlan , and B. C. Cowie , Surveillance for Hepatitis B Indicators: Tracking Australia's Progress Towards Hepatitis B Elimination – National Report 2022 (WHO Collaborating Centre for Viral Hepatitis, The Peter Doherty Institute for Infection and Immunity, 2022), accessed May 2025, https://www.doherty.edu.au/wp‐content/uploads/2026/01/National_Surveillance_for_Hepatitis_B_Indicators_final2.pdf.

[5] K. Hosking , T. De Santis , E. Vintour‐Cesar , et al., ““Putting the Power Back Into Community”: A Mixed Methods Evaluation of a Chronic Hepatitis B Training Course for the Aboriginal Health Workforce of Australia's Northern Territory,” PLoS One 19, no. 1 (2024): e0288577. 10.1371/journal.pone.0288577 PMC10807824 38266007

[6] K. Hosking , P. Binks , T. De Santis , et al., “Evaluating a Novel Model of Hepatitis B Care, Hep B PAST, in the Northern Territory of Australia: Results From a Prospective, Population‐Based Study,” Lancet Regional Health ‐ West Pacific 48 (2024): 101116. 10.1016/j.lanwpc.2024.101116 PMC11222935 38966601

[7] T. Hla , S. Bukulatjpi , P. Binks , G. Gurruwiwi , R. Dhurrkay , and J. Davies , “A “One Stop Liver Shop” Approach Improves the Cascade‐Of‐Care for Aboriginal and Torres Strait Islander Australians Living With Chronic Hepatitis B in the Northern Territory of Australia: Results of a Novel Care Delivery Model,” International Journal for Equity in Health 19, no. 1 (2020): 64. 10.1186/s12939-020-01180-w PMC7206741 32381081

[8] K. Hosking , T. De Santis , E. Vintour‐Cesar , et al., ““The Most Culturally Safe Training I've Ever Had”: The Co‐Design of a Culturally Safe Managing Hepatitis B Training Course With and for the Aboriginal Health Workforce of the Northern Territory of Australia,” BMC Health Services Research 23, no. 1 (2023): 935. 10.1186/s12913-023-09902-w PMC10472722 37653370

[9] Australasian Society for HIV and Viral Hepatitis and Sexual Health Medicine , Hepatitis B s100 Prescriber Program: A Guide for General Practitioners, Other Community‐Based Medical Practitioners and Nurse Practitioners (2020), accessed March 2025, https://ashm.org.au/wp‐content/uploads/2022/04/hbvprescriberhandbook2020.pdf.

[10] Australian Centre for Disease Control , Fourth National Hepatitis B Strategy 2025–2030 (Australian Centre for Disease Control, 2025), accessed May 2026, https://www.cdc.gov.au/sites/default/files/2026‐04/fourth‐national‐hepatitis‐b‐strategy‐2025‐2030_1.pdf.

[11] M. E. Drummond , M. J. Sculpher , G. W. Torrance , B. J. O'Brien , and G. L. Stoddart , Methods for the Economic Evaluation of Health Care Programmes, 3rd ed. (Oxford University Press, 2005), accessed August 2026, https://academic.oup.com/book/54294.

[12] A. Olsen , J. Wallace , and L. Maher , “Responding to Australia's National Hepatitis B Strategy 2010–13: Gaps in Knowledge and Practice in Relation to Indigenous Australians,” Australian Journal of Primary Health 20, no. 2 (2014): 134–142. 10.1071/PY12162 24216010

[13] Australian Bureau of Statistics , “Consumer Price Index, Australia: Australian Bureau of Statistics,” (2023), accessed July 2024, https://www.abs.gov.au/statistics/economy/price‐indexes‐and‐inflation/consumer‐price‐index‐australia/sep‐quarter‐2023.

[14] Australian Bureau of Statistics , Census of Population and Housing: General Community Profile, Northern Territory (Australian Bureau of Statistics, 2021), accessed April 2024, https://www.abs.gov.au/census/find‐census‐data/community‐profiles/2021/7.

[15] Independent Health and Aged Care Pricing Authority , National Efficient Cost Determination 2023–24 (Independent Health and Aged Care Pricing Authority, 2023), accessed August 2026, https://www.ihacpa.gov.au/resources/national‐efficient‐price‐determination‐2023‐24.

[16] Independent Health and Aged Care Pricing Authority , NWAU Calculators (Independent Health and Aged Care Pricing Authority, 2023), accessed June 2024, https://www.ihacpa.gov.au/health‐care/pricing/nwau‐calculators.

[17] Independent Hospital Pricing Authority , Australian Refined Diagnosis Related Groups Version 11.0 Final Report (Independent Hospital Pricing Authority, 2022), accessed June 2025, https://www.ihacpa.gov.au/sites/default/files/2022‐08/ar‐drg_v11.0_final_report.pdf.

[18] Independent Health and Aged Care Pricing Authority , Australian Emergency Care Classification Version 1.1 Final Report (Independent Health and Aged Care Pricing Authority, 2024), accessed June 2025, https://www.ihacpa.gov.au/sites/default/files/2024‐08/australian_emergency_care_classification_version_1.1_final_report.pdf.

[19] Independent Health and Aged Care Pricing Authority , Tier 2 Non‐Admitted Services Definitions Manual 2023–24 (2023), accessed March 2024, https://www.ihacpa.gov.au/sites/default/files/2023‐03/tier_2_non‐admitted_services_definition_manual_2023‐24_0.pdf.

[20] Y. Zhao , P. Hanssens , P. Byron , and S. Guthridge , Cost Estimates of Primary Health Care Activities for Remote Aboriginal Communities in the Northern Territory (Department of Health and Community Services, Northern Territory, 2006), accessed June, 2025, https://digitallibrary.health.nt.gov.au/entities/publication/ececc474‐a00c‐4dd1‐bb08‐4ad89c84d08e.

[21] Y. Zhao , D. J. Russell , S. Guthridge , et al., “Costs and Effects of Higher Turnover of Nurses and Aboriginal Health Practitioners and Higher Use of Short‐Term Nurses in Remote Australian Primary Care Services: An Observational Cohort Study,” BMJ Open 9, no. 2 (2019): e023906. 10.1136/bmjopen-2018-023906 PMC6398713 30787082

[22] Department of Health and Aged Care , PBS Publications Archive (Department of Health and Aged Care, 2024), accessed December 2024, https://www.pbs.gov.au/info/publication/schedule/archive.

[23] R Core Team , R: A Language and Environment for Statistical Computing (R Foundation for Statistical Computing, 2025), accessed March 2025, https://www.R‐project.org/.

[24] B. Derrick , D. Toher , B. Russ , and P. White , “Test Statistics for the Comparison of Means for Two Samples That Include Both Paired and Independent Observations,” Journal of Modern Applied Statistical Methods 16 (2017): 137–157.

[25] B. Derrick , A. Dobson‐Mckittrick , D. Toher , and P. White , “Test Statistics for Comparing Two Proportions With Partially Overlapping Samples,” Journal of Applied Quantitative Methods 10, no. 3 (2015): 1–14.

[26] Australian Institute of Health and Welfare , Contribution of Chronic Disease to the Gap in Mortality Between Aboriginal and Torres Strait Islander People and Other Australians (Australian Institute of Health and Welfare, 2011), accessed September 2024, https://www.aihw.gov.au/getmedia/79b73a27‐c970‐47f0‐931b‐32d7badade40/12304.pdf?v=20230605180941&inline=true.

[27] K. McCulloch , N. Romero , J. MacLachlan , N. Allard , and B. Cowie , “Modeling Progress Toward Elimination of Hepatitis B in Australia,” Hepatology 71, no. 4 (2020): 1170–1181. 10.1002/hep.30899 PMC7187332 31419332

[28] Y. Xiao , J. Howell , C. van Gemert , et al., “Enhancing the Hepatitis B Care Cascade in Australia: A Cost‐Effectiveness Model,” Journal of Viral Hepatitis 27, no. 5 (2020): 526–536. 10.1111/jvh.13252 31856377

[29] K. McCulloch , N. Romero , N. Allard , J. H. MacLachlan , and B. C. Cowie , “Modelling Jurisdictional Disparities in the Cascade of Care for Chronic Hepatitis B in Australia: Impact of Treatment Uptake on Mortality,” Australian and New Zealand Journal of Public Health 47, no. 1 (2023): 100011. 10.1016/j.anzjph.2022.100011 36638581

[30] A. L. T. Nguyen , C. L. Blizzard , K. C. Yee , J. A. Campbell , A. J. Palmer , and B. de Graaff , “Hospitalisation Costs of Primary Liver Cancer in Australia: Evidence From a Data‐Linkage Study,” Australian Health Review 46, no. 4 (2022): 463–470. 10.1071/AH21395 35584964

[31] Australian Government Department of Health , Schedule of Pharmaceutical Benefits: Effective 1 July 2014–31 July 2014 (Australian Government Department of Health, 2014), accessed May 2024, https://www.pbs.gov.au/publication/schedule/2014/07/2014‐07‐01‐general‐schedule.pdf?variant=3.

[32] Australian Government Department of Health and Aged Care , Schedule of Pharmaceutical Benefits. Section 100 – Items Available Under Special Arrangement – Volume 2. Effective 1 July 2023 (Australian Government Department of Health and Aged Care, 2023), accessed June 2025, https://www.pbs.gov.au/publication/schedule/2023/07/2023‐07‐01‐general‐schedule‐volume‐2.pdf?variant=3.

