# Supplementary material for: Costs of Delivering the Hep B PAST Model of Care in Remote Australia: A Retrospective Pre–Post Study
Source: Med J Aust. 2026 Sep 30;224(10):e70292. doi: 10.5694/mja2.70292 (PMC13624689; doi:10.5694/mja2.70292)
Supplement: Supplementary file 1 [file MJA2-224-0-s001.pdf]

The Medical Journal of Australia

Supporting Information

Supplementary material

This appendix was part of the submitted manuscript and has been peer reviewed.
It is posted as supplied by the authors.

Appendix to: H. Nguyen, A.L.T. Nguyen, P. Binks, et al. Costs of delivering the Hep B PAST model of care in remote Australia: a retrospective pre–post study. *Med J Aust* 2026; doi: 10.5694/mja2.70292.

Table of Contents

Part 1. Process for determining the final cohort **3**

Part 2. Supplementary tables **5**

Table S1. CHEERS 2022 Checklist *5*

Table S2. Principal and additional diagnosis codes (ICD-10-AM) for inpatient admissions and ED presentations included in the study *10*

Table S3. Unit costs and assumptions used in this study *12*

Table S4. Age group distribution of the study cohort pre-and post-Hep B PAST *14*

Table S5. Annual number of individuals, contacts, and mean/median costs per individual and per contact for each healthcare service pre-and post-Hep B PAST *15*

Table S6. Summary costs of CHB antiviral and other liver-related medications *20*

Table S7. Sensitivity analyses of total healthcare costs for outreach and primary care services pre-and post-Hep B PAST *21*

Part 3. References **23**

Part 1. Process for determining the final cohort

1. ***Matching usual clinic and implementation date to each individual***

To enable comparison of hospital costs pre-and post-Hep B PAST, each individual was matched to their usual clinic and the corresponding implementation date. Usual clinic information was determined using data from the PCIS, if unavailable, the nearest clinic to the individual's locality was assumed to be their usual clinic. The implementation date for each clinic was determined as the date when the HBV status of patients at that clinic were established and linked to their care package managed by the Hep B Hub, which is part of the Hep B PAST model of care activities. Ultimately, each patient was allocated a usual clinic and a specific time of entry (implementation date) into the Hep B PAST program. For example, if a patient participated in Hep B PAST in July 2020, the six-month period prior to their participation was considered the pre-Hep B PAST, while the following six months were considered the post-Hep B PAST. This approach was applied to all visits across all care types including inpatient, ED, outpatient, outreach, and primary care services and includes all prescriptions for antiviral and other liver-related medications.

2. Selection of care service related to liver diseases or its sequelae

For each individual, only inpatient episodes and emergency department presentations mentioning liver diseases or related to the underlying diagnosis of hepatocellular carcinoma or cirrhosis were included in the cost estimates. The research team (HN, BdG, AL) reviewed all principal and additional diagnosis codes in the format of International Classification of Diseases, Tenth Revision, Australian Modification (ICD-10-AM). For the cases in which a discrepancy was identified, two infectious disease physicians (JSD and JD) were involved to make a final determination. The selection process was performed on a total of 42,947 inpatient episodes (1,510 individuals), 18,575 emergency presentations (1,487 individuals). The list of principal and additional diagnosis codes selected for this study is listed in the Table S2.

For outpatient attendances, all the clinic names and the services provided to the patients were reviewed to determine which attendances related to liver diseases. The selection process was performed on a total of 1,748 individuals (44,319 attendances) by three co-authors (HN, BdG, AL). Outreach service data in the NT was sourced from the PCIS database, which recorded the number of outreach visits in the communities. As outreach data is concurrently recorded within the outpatient data system, three co-authors (HN, BdG, AL) conducted a comprehensive review of services to each patient to distinguish between outpatient and outreach services. Given that data from community visits might be entered into the system within a 14-day timeframe based on consultations with data entry staff, an assumption has been made that any outreach activities recorded within 14 days would be considered part of a single outreach visit.

Primary care data from the NT was retrieved from the PCIS database. All liver/CHB related attendants were selected by staff from the NT Department of Health and provided to the research team.

To reflect the actual costs of CHB in First Nations peoples in the NT, those who are not First Nations peoples (n=475) were excluded from the final cohort. Among the First Nation cohort, individuals with usual residence in Darwin and Palmerston (n=80) were excluded as these patients were involved in the Hep B PAST, but the healthcare services they received was similar to the care they had received previously, making cost comparisons for this group inaccurate. Individuals who were incarcerated during the study period (n=213) were excluded because their healthcare costs were not borne by NT Health. Furthermore, individuals from other states or overseas, or those with incomplete information (n=43), were also excluded from the study. The datasets initially included records from January 2014, however, the six-month period from January 2014 to June 2014 did not constitute a complete financial year. Therefore, to maintain comparability across financial years, data from this period were excluded. Additionally, 82 individuals who have no healthcare contacts or only contacts during 6-month wash out period were also not included in costing comparisons.

The Hep B PAST program was formally established in 2018, with progressive implementation across participating communities between 2018 and 2023.

However, the program was piloted in five remote communities (Gunbalanya, Jabiru, Maningrida, Minjilang [Croker Island], and Warruwi [Goulburn Island]) from 2016. In these pilot sites, healthcare contacts occurring after the commencement of Hep B PAST activities were classified as post-Hep B PAST implementation.

Part 2. Supplementary tables

Table S1. CHEERS 2022 Checklist

Topic	No.	Item	Location where item is reported
**Title**			
	1	Identify the study as an economic evaluation and specify the interventions being compared.	Title: "Hep B PAST model of care: Costs of delivering services in remote Australia" – identifies the study as an economic evaluation and specifies the intervention (Hep B PAST model of care) in comparison with usual care.
**Abstract**			
	2	Provide a structured summary that highlights context, key methods, results, and alternative analyses.	Abstract: Structured summary including Objectives, Study type, Setting, Participants, Main outcome measures, Results, and Conclusions, covering context, key methods, and study findings.
**Introduction**			
**Background and objectives**	3	Give the context for the study, the study question, and its practical relevance for decision making in policy or practice.	In the Introduction: Describes the study context, study question, and its practical relevance for decision-making in policy and practice.
**Methods**			
**Health economic analysis plan**	4	Indicate whether a health economic analysis plan was developed and where available.	The costing methodology and statistical analysis plan are described in the Methods section.
**Study population**	5	Describe characteristics of the study population (such as age range, demographics, socioeconomic, or clinical characteristics).	In the Methods (Setting and cohort) and Results (Table 1): Describe the study population, including First Nations individuals with chronic hepatitis B living in remote Northern Territory communities, with demographic characteristics. In Table S4, information on age and sex of the cohort is included.

Topic	No.	Item	Location where item is reported
**Setting and location**	6	Provide relevant contextual information that may influence findings.	Methods (Setting and cohort): Describes the study setting in remote Northern Territory communities, including geographic context, population characteristics, and implementation of the Hep B PAST program across clinics participated the program.
**Comparators**	7	Describe the interventions or strategies being compared and why chosen.	In the Methods (Pre-and post-design): Describes the comparators as pre-Hep B PAST implementation (usual care) and post-Hep B PAST implementation (Hep B PAST model of care), using a pre-post design to assess changes in healthcare costs.
**Perspective**	8	State the perspective(s) adopted by the study and why chosen.	Methods (Costing methodology): States that the analysis was conducted from a health system perspective (Northern Territory Department of Health and the Commonwealth), reflecting the primary payers of healthcare costs and chosen to inform decision-making on healthcare funding and resource allocation.
**Time horizon**	9	State the time horizon for the study and why appropriate.	Methods (Setting and cohort): The time horizon was 2014–2023, enabling comparison of healthcare costs pre-and post-Hep B PAST implementation and reflecting the maximum available data period for analysis.
**Discount rate**	10	Report the discount rate(s) and reason chosen.	Discounting was not applied as this study analysed observed costs over the study period (2014–2023), with all costs adjusted to 2023 Australian dollars using the Consumer Prices Index.
**Selection of outcomes**	11	Describe what outcomes were used as the measure(s) of benefit(s) and harm(s).	In the Methods, outcomes were healthcare costs, including annual mean and median costs per individual and per contact across different care services, used to compare pre-and post-Hep B PAST.
**Measurement of outcomes**	12	Describe how outcomes used to capture benefit(s) and harm(s) were measured.	Methods (Costing methodology): Healthcare costs were measured by identifying healthcare contacts across service types and aggregating costs per contact and per individual.

Topic	No.	Item	Location where item is reported
**Valuation of outcomes**	13	Describe the population and methods used to measure and value outcomes.	Methods (Costing methodology): Healthcare costs were estimated by applying unit costs to healthcare services using standard sources, including National Efficient Prices, Pharmaceutical Benefits Scheme data, and Northern Territory Department of Health report.
**Measurement and valuation of resources and costs**	14	Describe how costs were valued.	Methods (Costing methodology): Resource use was measured as healthcare contacts across service types, and costs were valued by applying unit costs from standard sources. A detailed summary of unit costs and assumptions used in this study is in Table S3.
**Currency, price date, and conversion**	15	Report the dates of the estimated resource quantities and unit costs, plus the currency and year of conversion.	Methods (Costing methodology): Resource use was measured over the period 2014–2023, with unit costs derived from each relevant years. All costs were expressed in 2023 Australian dollars (A$) and inflated using Consumer Price Index inflators.
**Rationale and description of model**	16	If modelling is used, describe in detail and why used. Report if the model is publicly available and where it can be accessed.	Not applicable. This study was based on observed data using a retrospective pre–post design.
**Analytics and assumptions**	17	Describe any methods for analysing or statistically transforming data, any extrapolation methods, and approaches for validating any model used.	Methods (Statistical analysis; Sensitivity analysis): Statistical analyses included descriptive statistics and partially overlapping t-tests to compare annual mean costs per individual between pre- and post-Hep B PAST. Sensitivity analyses were conducted to assess uncertainty in key cost inputs, including outreach and primary care costs, with assumptions and data sources detailed in the Methods and Table S3.
**Characterising heterogeneity**	18	Describe any methods used for estimating how the results of the study vary for subgroups.	Not applicable: No subgroup analyses were conducted. However, results are presented by service type to describe variation in cost pre- and post-Hep B PAST.

Topic	No.	Item	Location where item is reported
**Characterising distributional effects**	19	Describe how impacts are distributed across different individuals or adjustments made to reflect priority populations.	Not applicable: No distributional or equity analyses were conducted; results are presented for the overall study cohort, which comprises First Nations Peoples living with chronic hepatitis B in Northern Territory remote communities.
**Characterising uncertainty**	20	Describe methods to characterise any sources of uncertainty in the analysis.	Methods (Sensitivity analysis; Statistical analysis): Uncertainty was characterised through sensitivity analyses (including outreach and primary care costs), and by reporting mean costs with 95% confidence intervals, and reporting median costs with interquartile ranges. Statistical comparisons using partially overlapping t-tests.
**Approach to engagement with patients and others affected by the study**	21	Describe any approaches to engage patients or service recipients, the general public, communities, or stakeholders (such as clinicians or payers) in the design of the study.	Introduction: The Hep B PAST model of care was developed in response to First Nations community needs and incorporates engagement with communities and healthcare providers. The Indigenous Reference Group was involved in discussions around study design and interpretation of results.
**Results**			
**Study parameters**	22	Report all analytic inputs (such as values, ranges, references) including uncertainty or distributional assumptions.	Unit costs, data sources, and key assumptions (including outreach and primary care costs) are described in Methods (Costing methodology, Sensitivity analysis). The summary of unit costs and assumptions are presented in Table S3.
**Summary of main results**	23	Report the mean values for the main categories of costs and outcomes of interest and summarise them in the most appropriate overall measure.	Results: Report annual mean and median costs per individual across service types and overall, including 95% confidence intervals with comparisons between pre- and post-Hep B PAST.
**Effect of uncertainty**	24	Describe how uncertainty about analytic judgments, inputs, or projections affect findings. Report the effect of choice of discount rate and time horizon, if applicable.	Results: Variations in total costs (sensitivity analyses in variations in outreach and primary care costs) affected absolute cost estimates and mean cost ranges but did not materially change overall cost patterns or the interpretation of findings.

Topic	No.	Item	Location where item is reported
**Effect of engagement with patients and others affected by the study**	25	Report on any difference patient/service recipient, general public, community, or stakeholder involvement made to the approach or findings of the study	The results were presented to the Indigenous Reference Group: this did not result in differences in study findings.
**Discussion**			
**Study findings, limitations, generalisability, and current knowledge**	26	Report key findings, limitations, ethical or equity considerations not captured, and how these could affect patients, policy, or practice.	Discussion and Conclusions: A summary of key findings includes discussion that that the Hep B PAST model delivers enhanced CHB care with relatively similar overall costs compared with usual care, study limitations (including costing assumptions and generalisability) are discussed, and implications for policy and practice in improving access to care for First Nations populations in remote settings is highlighted.
**Other relevant information**			
**Source of funding**	27	Describe how the study was funded and any role of the funder in the identification, design, conduct, and reporting of the analysis	Funding sources and their role are described in the Ethics approval section, where it is stated that funders had no role in the study design, data collection, analysis, interpretation, or reporting.
**Conflicts of interest**	28	Report authors conflicts of interest according to journal or International Committee of Medical Journal Editors requirements.	Conflicts of interest are reported after the Acknowledgements section, where it is stated: "Competing interests: No relevant disclosures."

From: Husereau D, Drummond M, Augustovski F, et al. Consolidated Health Economic Evaluation Reporting Standards 2022 (CHEERS 2022) Explanation and Elaboration: A Report of the ISPOR CHEERS II Good Practices Task Force. Value Health 2022;25. doi:10.1016/j.jval.2021.10.008

Table S2. Principal and additional diagnosis codes (ICD-10-AM) for inpatient admissions and ED presentations included in the study

No	Principal Diagnosis Codes	Additional diagnosis code
1	R10.0: Acute abdomen	Any additional diagnosis code
2	R10.3: Pain localised to other parts of lower abdomen	Any additional diagnosis code
3	R10.4: Other and unspecified abdominal pain	Any additional diagnosis code
4	R19.8: Other specified symptoms and signs involving the digestive system and abdomen	Any additional diagnosis code
5	R19.89: Other specified symptoms and signs involving the digestive system and abdomen	Any additional diagnosis code
6	R63.0: Anorexia	Any additional diagnosis code
7	R63.4: Abnormal weight loss	Any additional diagnosis code
8	B16.1: Acute hepatitis B with delta-agent (coinfection) without hepatic coma	Any additional diagnosis code
9	B16.9: Acute hepatitis B without delta-agent and without hepatic coma	Any additional diagnosis code
10	B17.9: Acute viral hepatitis unspecified	Any additional diagnosis code
11	B18.1: Chronic viral hepatitis B without delta-agent	Any additional diagnosis code
12	B19.9: Unspecified viral hepatitis without hepatic coma	Any additional diagnosis code
13	C22.0: Liver cell carcinoma	Any additional diagnosis code
14	C22.1: Intrahepatic bile duct carcinoma	Any additional diagnosis code
15	C22.9: Malignant neoplasm of liver, unspecified	Any additional diagnosis code
16	C78.7: Secondary malignant neoplasm of liver and intrahepatic bile duct	Any additional diagnosis code
17	K70.1: Alcoholic hepatitis	Any additional diagnosis code
18	K70.3: Alcoholic cirrhosis of liver	Any additional diagnosis code
19	K70.4: Alcoholic hepatic failure	Any additional diagnosis code
20	K71.1: Toxic liver disease with hepatic necrosis	Any additional diagnosis code
21	K72.0: Acute and subacute hepatic failure	Any additional diagnosis code
22	K72.1: Chronic hepatic failure	Any additional diagnosis code
23	K72.9: Hepatic failure, unspecified	Any additional diagnosis code
24	K73.2: Chronic active hepatitis, not elsewhere classified	Any additional diagnosis code
25	K73.9: Chronic hepatitis, unspecified	Any additional diagnosis code
26	K74.6: Other and unspecified cirrhosis of liver	Any additional diagnosis code
27	K75.0: Abscess of liver	Any additional diagnosis code
28	K75.9: Inflammatory liver disease, unspecified	Any additional diagnosis code
29	K76.6: Portal hypertension	Any additional diagnosis code
30	K76.7: Hepatorenal syndrome	Any additional diagnosis code
31	K76.8: Other specified diseases of liver	Any additional diagnosis code
32	K76.9: Liver disease, unspecified	Any additional diagnosis code
33	K80.00: Calculus of gallbladder with acute cholecystitis, without mention of obstruction	Any additional diagnosis code
34	K80.01: Calculus of gallbladder with acute cholecystitis, with obstruction	Any additional diagnosis code
35	K80.10: Calc gallb w oth cholecystitis wo obs	Any additional diagnosis code
36	K80.11: Calculus of gallbladder with other cholecystitis, with obstruction	Any additional diagnosis code
37	K80.20: Calculus of gallbladder without cholecystitis, without mention of obstruction	Any additional diagnosis code
38	K80.30: Calculus of bile duct with cholangitis, without mention of obstruction	Any additional diagnosis code
39	K80.31: Calculus of bile duct with cholangitis, with obstruction	Any additional diagnosis code
40	K80.40: Calculus of bile duct with cholecystitis, without mention of obstruction	Any additional diagnosis code
41	K80.41: Calculus of bile duct with cholecystitis, with obstruction	Any additional diagnosis code
42	K80.50: Calculus of bile duct without cholangitis or cholecystitis, without mention of obstruction	Any additional diagnosis code
43	K81.0: Acute cholecystitis	Any additional diagnosis code
44	K81.1: Chronic cholecystitis	Any additional diagnosis code
45	K81.9: Cholecystitis, unspecified	Any additional diagnosis code
46	K82.8: Other specified diseases of gallbladder	Any additional diagnosis code
47	K83.0: Cholangitis	Any additional diagnosis code
48	K83.9: Disease of biliary tract, unspecified	Any additional diagnosis code
49	K85.1: Biliary acute pancreatitis	Any additional diagnosis code
50	K85.2: Alcohol-induced acute pancreatitis	Any additional diagnosis code
51	K85.9: Acute pancreatitis, unspecified	Any additional diagnosis code
52	K86.0: Alcohol-induced chronic pancreatitis	Any additional diagnosis code

No	Principal Diagnosis Codes	Additional diagnosis code
53	K86.1: Other chronic pancreatitis	Any additional diagnosis code
54	K86.9: Disease of pancreas, unspecified	Any additional diagnosis code
55	O98.4: Viral hepatitis in pregnancy, childbirth and the puerperium	Any additional diagnosis code
56	R11: Nausea and vomiting	Any additional diagnosis code
57	R12: Heartburn	Any additional diagnosis code
58	R14: Flatulence and related conditions	Any additional diagnosis code
59	R16.0: Hepatomegaly, not elsewhere classified	Any additional diagnosis code
60	R17: Unspecified jaundice	Any additional diagnosis code
61	R18: Ascites	Any additional diagnosis code
62	S36.10: Injury of liver, unspecified	Any additional diagnosis code
63	S36.11: Contusion and haematoma of liver	Any additional diagnosis code
64	S36.14: Moderate laceration of liver	Any additional diagnosis code
65	A40.1: Sepsis due to streptococcus, group B	C22.0 Liver cell carcinoma
66	R45.89: Other symptoms and signs involving emotional state	C22.0 Liver cell carcinoma
67	A24.4: Melioidosis unspecified	K70.3 Alcoholic cirrhosis of liver
68	A40.1: Sepsis due to streptococcus, group B	K70.3 Alcoholic cirrhosis of liver
69	A41.51: Sepsis due to Escherichia coli [E. Coli]	K70.3 Alcoholic cirrhosis of liver
70	A41.58: Sepsis due to other Gram-negative organisms	K70.3 Alcoholic cirrhosis of liver
71	A49.11: Streptococcal infection, unspecified site	K70.3 Alcoholic cirrhosis of liver
72	M00.22: Other streptococcal arthritis and polyarthritis, upper arm	K70.3 Alcoholic cirrhosis of liver
73	A40.2: Sepsis due to streptococcus, group D and enterococcus	K74.6: Other and unspecified cirrhosis of liver
74	A40.3: Sepsis due to Streptococcus pneumoniae	K74.6: Other and unspecified cirrhosis of liver
75	A41.51: Sepsis due to Escherichia coli [E. Coli]	K74.6: Other and unspecified cirrhosis of liver
76	A41.9: Sepsis, unspecified	K74.6: Other and unspecified cirrhosis of liver
77	A49.8: Other bacterial infections of unspecified site	K74.6: Other and unspecified cirrhosis of liver
78	J13: Pneumonia due to Streptococcus pneumoniae	K74.6: Other and unspecified cirrhosis of liver
79	R45.89: Other symptoms and signs involving emotional state	K74.6: Other and unspecified cirrhosis of liver

Table S3. Unit costs and assumptions used in this study

Care Services	Unit cost (A$)	Classification	Assumption
Inpatient services	National Weighted Activity Unit (NWAU) [1] × National Efficient Price (NEP) per inpatient admission [2]	Australian Refined Diagnosis Related Groups classifications [3]	Costs were calculated using year-specific NEP and NWAU values and inflated to 2023–24 Australian dollars (A$) using the Consumer Price Index [4].
Emergency department	NWAU [1] × NEP per ED presentation [5]	Urgency Related Groups [5] (year: 2014–2020), Urgency Disposition Groups [5] (year: 2021–2023)	Costs were calculated using year-specific NEP and NWAU values and inflated to 2023–24 Australian dollars (A$) using Consumer Price Index [4].
Outpatient services	NWAU [1] × NEP per outpatient visit [6]	Tier 2 Non-Admitted Services Classification [6]	Costs were calculated using year-specific NEP and NWAU values and inflated to 2023–24 Australian dollars (A$) using the Consumer Price Index [4].<br>Travel cost assumed A$1/km based on literature [7,8].
Primary care services	A$266 (year: 2017–18); A$256 (year: 2019–20); A$226 (year: 2021–22)	Not applicable	Primary care unit costs were available from internal NT Department of Health data for three years: A$266 in 2017–18, A$256 in 2019–20, and A$226 in 2021–22.<br>For other years, unit costs were estimated using Consumer Price Index-based adjustments anchored to the nearest available year. Specifically, costs for 2013–14 to 2016–17 and 2018–19 were derived by adjusting the 2017–18 estimate, while costs for 2020–21 were based on the 2019–20 estimate, and costs for 2022–23 were based on the 2021–22 estimate.
Outreach services	A$450 per visit in 2023–24	Not applicable	It was assumed that each outreach visit involved 15 individuals per clinic, based on observations from service providers delivering the outreach program.<br>The per-patient cost of A$450 per visit was derived from program budgeting documents of the Hep B PAST project for the 2023–24 financial year. This cost encompassed a range of expenses, including salaries, accommodation, allowances, charter flights to remote communities, and local transportation

			rentals. Costs for other years were deflated using Consumer Price Index [4].
Antiviral medications	Pharmaceutical Benefits Scheme (PBS)-listed price (year-specific) per prescription [9]	Not applicable	Extracted from PBS [9] and inflated to 2023–24 A$ using Consumer Price Index [4].
Other medications	PBS-listed price (year-specific) per prescription [9]	Not applicable	Included if CHB-related based on clinician review.
Sensitivity analysis (Outreach)	A$400 per patient per outreach visit in 2023–24	Not applicable	Outreach service at A$400 per patient per visit
Sensitivity analysis (Outreach)	A$500 per patient per outreach visit in 2023–24	Not applicable	Outreach service at A$500 per patient per visit
Sensitivity analysis (Primary care services)	±A$5 annual change for primary care service	Not applicable	Primary care unit costs showed a decreasing trend over the observed period, from A$266 in 2017–18, A$256 in 2019–2020, and A$226 in 2021–2022. In the absence of data for other years, unit costs were estimated by applying a consistent annual change of A$5, reflecting this observed trend. Specifically, costs were adjusted upwards by A$5 per year for earlier years and downwards by A$5 per year for later years relative to the closest available data point.<br>This resulted in estimated unit costs of A$286 (2013–14), A$281 (2014–15), A$276 (2015–16), A$271 (2016–17), A$261 (2018–19), A$251 (2020–21), and A$221 (2022–23).

Table S4. Age group distribution of the study cohort pre-and post-Hep B PAST

	Pre-Hep B PAST			Post-Hep B PAST		
	**Total (n)**	**Male (n, %)**	**Female (n, %)**	**Total (n)**	**Male (n, %)**	**Female (n, %)**
**Age (years)**						
<10	n/r	n/r	n/r	0	0	0
10–19	18	10 (56)	8 (44)	n/r	n/r	n/r
20–29	65	42 (65)	23 (35)	38	24 (63)	14 (37)
30–39	281	158 (56)	123 (44)	163	89 (55)	74 (45)
40–49	287	153 (53)	134 (47)	289	158 (55)	131 (45)
50–59	221	140 (63)	81 (37)	258	155 (60)	103 (40)
60–69	123	59 (48)	64 (52)	198	112 (57)	86 (43)
70–79	47	24 (51)	23 (49)	78	37 (78)	41 (53)
80–89	13	n/r	n/r	22	9 (22)	13 (59)
90+	n/r	n/r	n/r	n/r	n/r	n/r
Median Age (IQR)	46 (36–56)	46 (36–56)	45 (37–57)	51 (42–62)	51 (41–61)	51 (42–63)

Note: n/r indicates data not reported due to confidentiality requirements where counts are less than or equal 5.

Table S5. Annual number of individuals, contacts, and mean/median costs per individual and per contact for each healthcare service pre-and post-Hep B PAST

Service Type	Financial Year	Pre-Hep B PAST						Post-Hep B PAST					
		Number of Individual (Contact)	Total Cost (Pre)	Mean cost/ Contact/year (95% CI)	Median cost/ Contact/year (IQR)	Mean cost/ Individual/year (95% CI)	Median cost/ Individual/ year (IQR)	Number of Individual (Contact)	Total Cost (Post)	Mean cost/ Contact/year (95% CI)	Median cost/ Contact/year (IQR)	Mean cost/ Individual/year (95% CI)	Median cost/ Individual/ year (IQR)
Inpatient services	2014/15	18 (25)	221,778	8,871 (5,877; 11,865)	6,445 (4,934; 11,663)	12,321 (7,020; 17,622)	7,899 (5,676; 16,068)	0	0	0	0	0	0
Inpatient services	2015/16	18 (27)	256,433	9,498 (3,653; 15,342)	5,811 (1,679; 9,085)	14,246 (4,854; 23,639)	7,028 (2,183; 21,056)	0	0	0	0	0	0
Inpatient services	2016/17	21 (26)	303,185	11,661 (6,371; 16,951)	8,519 (1,757; 16,318)	14,437 (7,158; 21,717)	9,520 (1,618; 19,972)	n/r	n/r	n/r	n/r	n/r	n/r
Inpatient services	2017/18	21 (28)	215,658	7,702 (5,275; 10,130)	6,746 (1,869; 10,637)	10,269 (6,866; 13,673)	9,046 (5,598; 15,361)	n/r	n/r	n/r	n/r	n/r	n/r
Inpatient services	2018/19	11 (12)	153,421	12,785 (1,907; 23,663)	5,427 (2,306; 12,848)	13,947 (2,113; 25,782)	6,011 (3,144; 14,746)	9 (11)	200,763	18,251 (5,760; 30,743)	9,486 (2,048; 32,364)	22,307 (2,668; 41,946)	9,486 (2,079; 33,360)
Inpatient services	2019/20	n/r	130,660	26,132 (-14,381; 66,645)	8,237 (7,777; 26,206)	32,665 (-30,755; 96,085)	16,992 (7,291; 42,365)	20 (31)	296,632	9,569 (4,989; 14,149)	5,190 (1,820; 10,957)	14,832 (6,470; 23,193)	8,551 (1,684; 18,346)
Inpatient services	2020/21	0	0	0	0	0	0	24 (55)	488,676	8,885 (6,695; 11,075)	6,783 (2,917; 12,043)	20,362 (10,140; 30,583)	12,640 (2,595; 27,538)
Inpatient services	2021/22	0	0	0	0	0	0	29 (37)	399,966	10,810 (5,584; 16,036)	3,036 (2,538; 14,561)	13,792 (6,802; 20,782)	8,842 (2,543; 16,944)
Inpatient services	2022/23	0	0	0	0	0	0	25 (38)	289,467	7,618 (4,446; 10,789)	4,088 (2,013; 8,625)	11,579 (4,423; 18,734)	3,866 (1,839; 8,934)
**Inpatient services (Total)**		**71 (123)**	**1,281,135**	**10,416 (8,052; 112,780)**	**6,758 (2,159; 12,236)**	**13,776 (10,461; 17,091)**	**7,777 (4,555; 18,274)**	**91 (176)**	**1,747,740**	**9,930 (8,088 11,773)**	**5,376 (2,401; 11,724)**	**15,745 (11,964; 19,527)**	**7,795 (2,120; 19,813)**
ED services	2014/15	42 (56)	70,777	1,264 (1,150; 1,378)	1,304 (982; 1,597)	1,685 (1,286; 2,084)	1,411 (1,143; 8,069)	0	0	0	0	0	0
ED services	2015/16	34 (48)	59,879	1,247 (1,143; 1,352)	1,379 (1,147; 1,492)	1,761 (1,289; 2,233)	1,311 (793; 5,508)	0	0	0	0	0	0
ED services	2016/17	43 (60)	65,255	1,088 (1,002; 1,173)	1,174 (957; 1,330)	1,518 (1,288; 1,748)	1,344 (1,036; 4,217)	n/r	n/r	n/r	n/r	n/r	n/r
ED services	2017/18	34 (55)	58,691	1,067	1,026	1,726	1,144	n/r	n/r	n/r	n/r	n/r	n/r

Service Type	Financial Year	Pre-Hep B PAST						Post-Hep B PAST					
		Number of Individual (Contact)	Total Cost (Pre)	Mean cost/ Contact/year (95% CI)	Median cost/ Contact/year (IQR)	Mean cost/ Individual/year (95% CI)	Median cost/ Individual/ year (IQR)	Number of Individual (Contact)	Total Cost (Post)	Mean cost/ Contact/year (95% CI)	Median cost/ Contact/year (IQR)	Mean cost/ Individual/year (95% CI)	Median cost/ Individual/ year (IQR)
				(982; 1,152)	(911; 1,327)	(1,007; 2,445)	(1,009; 11,329)						
ED services	2018/19	20 (27)	35,034	1,298 (1,173; 1,422)	1,308 (1,094; 1,420)	1,752 (1,298; 2,206)	1,420 (1,260; 5,063)	7 (8)	9,293	1,162 (949; 1,375)	1,126 (966; 1,404)	1,328 (783; 1,873)	1,122 (965; 2,533)
ED services	2019/20	n/r	10,239	1,463 (1,166; 1,760)	1,452 (1,256; 1,662)	2,048 (621; 3,475)	1,826 (1,452; 4,011)	33 (62)	83,453	1,346 (1,246; 1,446)	1,427 (1,122; 1,595)	2,529 (1,427; 3,630)	1,574 (1,287; 13,628)
ED services	2020/21	0	0	0	0	0	0	39 (82)	114,717	1,399 (1,317; 1,481)	1,532 (1,126; 1,618)	2,941 (1,869; 4,014)	1,680 (1,193; 15,424)
ED services	2021/22	0	0	0	0	0	0	48 (83)	121,275	1,461 (1,380; 1,542)	1,508 (1,162; 1,722)	2,527 (1,898; 3,155)	1,780 (1,496; 12,555)
ED services	2022/23	0	0	0	0	0	0	36 (85)	113,937	1,340 (1,232; 1,449)	1,260 (937; 1,714)	3,165 (1,887; 4,443)	1,780 (1,220; 20,431)
**ED services (Total)**		**126 (253)**	**299,876**	**1,185 (1,140; 1,231)**	**1,222 (1,002; 1,490)**	**1,685 (1,487; 1,882)**	**1,339 (1,032; 1,818)**	**125 (324)**	**447,432**	**1,381 (1,336; 1,426)**	**1,467 (1,090; 1,682)**	**2,679 (2,223; 3,136)**	**1,672 (1,246; 2,668)**
Outpatient services	2014/15	22 (59)	55,525	941 (757; 1,125)	469 (466; 1,510)	2,524 (1,127; 3,920)	1,953 (1,137; 2,305)	0	0	0	0	0	0
Outpatient services	2015/16	78 (153)	295,860	1,934 (1,813; 2,055)	2,032 (1,579; 2,343)	3,793 (1,422; 6,164)	1,857 (1,579; 2,045)	0	0	0	0	0	0
Outpatient services	2016/17	125 (213)	311,124	1,461 (1,372; 1,550)	1,652 (1,024; 1,985)	2,489 (1,869; 3,109)	1,985 (1,652; 1,986)	n/r	n/r	n/r	n/r	n/r	n/r
Outpatient services	2017/18	41 (75)	109,898	1,465 (1,313; 1,617)	1,463 (1,188; 1,996)	2,680 (1,392; 3,969)	1,490 (1,186; 2,300)	18 (49)	50,564	1,032 (941; 1,123)	871 (799; 1,149)	2,809 (-10; 5,628)	1,454 (1,061; 1,662)
Outpatient services	2018/19	11 (23)	30,800	1,339 (1,052; 1,626)	1,582 (616; 1,833)	2,800 (281; 5,319)	671 (555; 3,424)	24 (88)	79,372	902 (787; 1,017)	730 (611; 1,019)	3,307 (1,370; 5,244)	1,323 (564; 4,315)
Outpatient services	2019/20	8 (26)	43,751	1,683 (1,438; 1,927)	1,888 (1,081; 1,889)	5,469 (35; 10,903)	2,798 (2,103; 5,435)	49 (115)	121,934	1,060 (944; 1,177)	934 (615; 1,241)	2,488 (1,429; 3,548)	1,330 (1,062; 2,397)
Outpatient services	2020/21	0	0	0	0	0	0	50 (252)	375,455	1,490 (1,394; 1,586)	1,335 (994; 2,000)	7,509 (2,587; 12,431)	1,753 (959; 4,206)
Outpatient services	2021/22	0	0	0	0	0	0	61 (301)	365,029	1,213 (1,142; 1,283)	1,127 (636; 1,927)	5,984 (2,729; 9,239)	2,288 (1,558; 4,635)
Outpatient services	2022/23	0	0	0	0	0	0	42 (182)	231,708	1,273 (1,187; 1,359)	1,147 (949; 1,643)	5,517 (2,885; 8,148)	2,326 (1,085; 3,850)
**Outpatient services (Total)**		**222 (549)**	**846,957**	**1,543 (1,480;1,606)**	**1,659 (1,005;2,032)**	**2,972 (2,230; 3,713)**	**1,985 (1,458; 2,054)**	**149 (988)**	**1,224,660**	**1,240 (1,198; 1,281)**	**1,089 (750; 1,859)**	**4,999 (3,606; 6,391)**	**1,858 (1,045; 3,584)**
Outreach services	2014/15	380 (620)	220,720	356 (366; 366)	n/a	581 (545; 616)	356 (356; 712)	0	0	0	0	0	0
Outreach services	2015/16	397 (677)	245,074	362 (362; 362)	n/a	617 (583; 651)	362 (362; 724)	0	0	0	0	0	0
Outreach services	2016/17	381 (625)	228,750	366 (366; 366)	n/a	600 (564; 637)	366 (366; 732)	26 (28)	10,248	366 (366; 366)	n/a	394 (354; 434)	366 (366; 366)
Outreach services	2017/18	336 (505)	188,365	373 (373; 373)	n/a	561 (529; 593)	373 (373; 746)	100 (223)	83,179	373 (373; 373)	n/a	832 (735; 929)	746 (373; 1,119)

Service Type	Financial Year	Pre-Hep B PAST						Post-Hep B PAST					
		Number of Individual (Contact)	Total Cost (Pre)	Mean cost/ Contact/year (95% CI)	Median cost/ Contact/year (IQR)	Mean cost/ Individual/year (95% CI)	Median cost/ Individual/ year (IQR)	Number of Individual (Contact)	Total Cost (Post)	Mean cost/ Contact/year (95% CI)	Median cost/ Contact/year (IQR)	Mean cost/ Individual/year (95% CI)	Median cost/ Individual/ year (IQR)
Outreach services	2018/19	146 (211)	80,180	380 (380; 380)	n/a	549 (502; 596)	380 (380; 760)	258 (483)	183,540	380 (380; 380)	n/a	711 (661; 762)	760 (380; 760)
Outreach services	2019/20	102 (129)	49,794	386 (386; 386)	n/a	488 (442; 535)	386 (386; 386)	449 (943)	363,998	386 (386; 386)	n/a	811 (760; 861)	772 (386; 1,158)
Outreach services	2020/21	0	0	0	0	0	0	564 (1,156)	445,060	385 (385; 385)	n/a	789 (745; 833)	770 (385; 1,155)
Outreach services	2021/22	0	0	0	0	0	0	587 (1,043)	417,200	400 (400; 400)	n/a	711 (676; 745)	400 (400; 800)
Outreach services	2022/23	0	0	0	0	0	0	518 (887)	376,088	424 (424; 424)	n/a	726 (689; 763)	424 (424; 848)
**Outreach services (Total)**		**770 (2,767)**	**1,012,883**	**366 (366; 366)**	**n/a**	**581 (566; 597)**	**373 (362; 732)**	**856 (4,763)**	**1,879,313**	**395 (394; 395)**	**n/a**	**751 (732; 770)**	**746 (386; 848)**
Primary care services	2014/15	87 (111)	36,209	326 (326; 326)	n/a	416 (374; 458)	326 (325; 327)	0	0	0	0	0	0
Primary care services	2015/16	104 (135)	44,101	327 (327; 327)	n/a	424 (374; 474)	327 (326; 328)	0	0	0	0	0	0
Primary care services	2016/17	78 (104)	33,924	326 (326; 326)	n/a	435 (376; 494)	327 (326; 327)	n/r	1,960	327 (326; 327)	n/a	392 (211; 574)	327 (326; 327)
Primary care services	2017/18	61 (82)	26,408	322 (321; 323)	n/a	433 (366; 500)	325 (322; 325)	38 (48)	15,406	321 (319; 323)	n/a	405 (338; 473)	325 (323; 325)
Primary care services	2018/19	17 (27)	8,171	303 (302; 303)	n/a	481 (315; 646)	303 (302; 605)	79 (100)	30,291	303 (303; 303)	n/a	383 (343; 424)	303 (302; 303)
Primary care services	2019/20	15 (17)	5,107	300 (300; 301)	n/a	340 (281; 399)	300 (300; 302)	274 (381)	113,856	299 (298; 300)	n/a	416 (389; 443)	305 (300; 494)
Primary care services	2020/21	0	0	0	0	0	0	197 (309)	79,540	257 (257; 258)	n/a	404 (365; 442)	259 (257; 515)
Primary care services	2021/22	0	0	0	0	0	0	174 (241)	60,394	251 (250; 251)	n/a	347 (319; 375)	254 (248; 492)
Primary care services	2022/23	0	0	0	0	0	0	150 (236)	57,984	246 (245; 246)	n/a	387 (347; 426)	248 (244; 491)
**Primary care services (Total)**		**287 (476)**	**153,919**	**323 (323; 324)**	**n/a**	**425 (400; 450)**	**327 (325; 327)**	**516 (1,321)**	**359,432**	**272 (271; 273)**	**n/a**	**392 (377; 407)**	**302 (257; 492)**
Antiviral medications	2014/15	25 (34)	115,218	3,389 (2,479; 4,299)	3,096 (1,468; 4,879)	4,609 (3,585; 5,633)	4,477 (2,985; 6,261)	0	0	0	0	0	0
Antiviral medications	2015/16	35 (43)	145,296	3,379 (2,623; 4,135)	3,425 (1,197; 5,416)	4,151 (3,295; 5,008)	5,163 (1,957; 6,175)	0	0	0	0	0	0
Antiviral medications	2016/17	36 (41)	145,003	3,537 (2,819; 4,255)	3,382 (1,612; 5,768)	4,028 (3,325; 4,730)	4,591 (2,264; 5,768)	14 (16)	14,951	934 (729; 1,140)	964 (964; 964)	1,068 (833; 1,303)	964 (964; 964)
Antiviral medications	2017/18	38 (41)	99,877	2,436 (1,844; 3,028)	2,010 (822; 4,764)	2,628 (2,030; 3,227)	2,010 (1,491; 4,764)	22 (25)	99,003	3,960 (3,279; 4,642)	4,764 (3,153; 4,764)	4,500 (3,890; 5,110)	4,764 (3,364; 4,764)

Service Type	Financial Year	Pre-Hep B PAST						Post-Hep B PAST					
		**Number of Individual (Contact)**	**Total Cost (Pre)**	**Mean cost/ Contact/year (95% CI)**	**Median cost/ Contact/year (IQR)**	**Mean cost/ Individual/year (95% CI)**	**Median cost/ Individual/ year (IQR)**	**Number of Individual (Contact)**	**Total Cost (Post)**	**Mean cost/ Contact/year (95% CI)**	**Median cost/ Contact/year (IQR)**	**Mean cost/ Individual/year (95% CI)**	**Median cost/ Individual/ year (IQR)**
Antiviral medications	2018/19	21 (21)	19,281	918 (359; 1,477)	315 (315; 581)	918 (359; 1,477)	315 (315; 581)	60 (67)	163,911	2,446 (2,132; 2,761)	2,088 (1,780; 3,594)	2,732 (2,410; 3,054)	2,088 (2,088; 3,594)
Antiviral medications	2019/20	7 (7)	7,210	1,030 (665; 1,395)	1,078 (1,001; 1,187)	1,030 (665; 1,395)	1,078 (1,001; 1,187)	93 (101)	223,762	2,215 (1,948; 2,483)	2,562 (1,330; 2,562)	2,406 (2,121; 2,691)	2,562 (1,834; 2,562)
Antiviral medications	2020/21	0	0	0	0	0	0	116 (128)	200,965	1,570 (1,354; 1,786)	1,836 (709; 1,836)	1,732 (1,497; 1,968)	1,836 (984; 1,836)
Antiviral medications	2021/22	0	0	0	0	0	0	133 (147)	126,158	858 (739; 978)	930 (329; 930)	949 (824; 1,073)	930 (589; 930)
Antiviral medications	2022/23	0	0	0	0	0	0	133 (144)	107,333	745 (635; 856)	673 (274; 792)	807 (692; 922)	778 (381; 792)
**Antiviral medications (Total)**		**61 (187)**	**531,885**	**2,844 (2,509; 3,180)**	**2,039 (823; 4,764)**	**3,283 (2,912; 3,655)**	**2,766 (1,204; 5,342)**	**161(628)**	**936,084**	**1,491 (1,386; 1,595)**	**930 (583; 2,088)**	**1,639 (1,525; 1,753)**	**964 (741; 2,426)**
Other medications	2014/15	64 (104)	3,919	38 (27; 48)	14 (5; 50)	61 (43; 79)	31 (7; 84)	0	0	0	0	0	0
Other medications	2015/16	73 (123)	4,785	39 (31; 47)	22 (6; 53)	66 (48; 83)	42 (6; 98)	0	0	0	0	0	0
Other medications	2016/17	65 (100)	3,642	36 (26; 46)	15 (6; 47)	56 (38; 75)	15 (6; 79)	0	0	0	0	0	0
Other medications	2017/18	60 (90)	3,568	40 (27; 52)	18 (6; 52)	59 (36; 83)	21 (6; 66)	10 (12)	118	10 (5; 15)	6 (5; 10)	12 (6; 17)	10 (6; 11)
Other medications	2018/19	22 (29)	472	16 (9; 24)	10 (6; 12)	21 (12; 31)	11 (8; 32)	42 (50)	1,440	29 (18; 40)	6 (6; 40)	34 (21; 47)	10 (6; 61)
Other medications	2019/20	n/r	n/r	n/r	n/r	n/r	n/r	82 (133)	13,742	103 (9; 198)	11 (6; 61)	168 (10; 325)	26 (6; 89)
Other medications	2020/21	0	0	0	0	0	0	97 (170)	16,115	95 (7; 182)	9 (6; 45)	166 (10; 323)	18 (6; 96)
Other medications	2021/22	0	0	0	0	0	0	86 (144)	20,371	141 (24; 259)	28 (6; 82)	237 (35; 439)	41 (6; 144)
Other medications	2022/23	0	0	0	0	0	0	91 (167)	14,633	88 (14; 161)	19 (14; 161)	161 (22; 299)	43 (6; 107)
**Other medications (Total)**		**162 (450)**	**16,494**	**37 (32; 41)**	**16 (6; 51)**	**57 (49; 66)**	**26 (6; 80)**	**216 (676)**	**66,419**	**98 (56; 140)**	**13 (6; 58)**	**163 (92; 234)**	**24 (6; 96)**
All services (excl. Antivirals)	2014/15	436 (975)	601,146	617 (504; 729)	356 (356; 356)	1,379 (971; 1,786)	613 (356; 1,068)	0	0	0	0	0	0
All services (excl. Antivirals)	2015/16	519 (1,163)	897,114	771 (618; 925)	362 (362; 362)	1,729 (1,186; 2,271)	724 (362; 1,243)	0	0	0	0	0	0
All services (excl. Antivirals)	2016/17	458 (1,128)	939,427	833 (680; 986)	366 (366; 392)	2,051 (1,570; 2,532)	732 (366; 2,118)	28 (37)	65,132	1,760 (-1,051; 4,572)	366 (366; 366)	2,326 (-1,525; 6,178)	366 (366; 366)

Service Type	Financial Year	Pre-Hep B PAST						Post-Hep B PAST					
		**Number of Individual (Contact)**	**Total Cost (Pre)**	**Mean cost/ Contact/year (95% CI)**	**Median cost/ Contact/year (IQR)**	**Mean cost/ Individual/year (95% CI)**	**Median cost/ Individual/ year (IQR)**	**Number of Individual (Contact)**	**Total Cost (Post)**	**Mean cost/ Contact/year (95% CI)**	**Median cost/ Contact/year (IQR)**	**Mean cost/ Individual/year (95% CI)**	**Median cost/ Individual/ year (IQR)**
All services (excl. Antivirals)	2017/18	405 (835)	597,922	716 (595; 837)	373 (373; 373)	1,476 (1,105; 1,848)	373 (373; 1,012)	106 (338)	170,847	505 (425; 586)	373 (373; 373)	1,612 (1,047; 2,177)	746 (444; 1,712)
All services (excl. Antivirals)	2018/19	178 (329)	306,752	932 (508; 1,357)	380 (380; 380)	1,723 (875; 2,572)	380 (380; 1,101)	292 (740)	499,778	675 (454; 897)	380 (380; 380)	1,712 (1,005; 2,419)	760 (380; 1,140)
All services (excl. Antivirals)	2019/20	110 (188)	238,905	1,271 (360; 2,181)	386 (386; 386)	2,172 (30; 4,314)	386 (386; 610)	566 (1,665)	976,296	586 (485; 688)	386 (256; 386)	1,725 (1,295; 2,155)	772 (386; 1,282)
All services (excl. Antivirals)	2020/21	0	0	0	0	0	0	632 (2,024)	1,508,739	745 (659; 831)	385 (385; 385)	2,387 (1,684; 3,090)	770 (385; 1,444)
All services (excl. Antivirals)	2021/22	0	0	0	0	0	0	666 (1,849)	1,378,379	745 (624; 867)	400 (400; 400)	2,070 (1,550; 2,589)	800 (400; 1,256)
All services (excl. Antivirals)	2022/23	0	0	0	0	0	0	594 (1,595)	1,081,775	678 (586; 771)	424 (242; 424)	1,821 (1,338; 2,304)	680 (424; 1,253)
**All services (excl. Antivirals)**		**859 (4,618)**	**3,581,264**	**776 (697; 854)**	**366 (356; 380)**	**1,701 (1,459; 1,942)**	**639 (366; 1,156)**	**926 (8,248)**	**5,680,946**	**689 (639; 739)**	**386 (366; 424)**	**1,970 (1,721; 2,218)**	**770 (400; 1,272)**

Note: n/r indicates data not reported due to confidentiality requirements where counts are less than or equal 5.
n/a indicates not applicable; for outreach and primary services, a single average cost per visit was applied and median and IQR values per visit are not appropriate.

Table S6. Summary costs of CHB antiviral and other liver-related medications

The costs of all medications were extracted from the PBS according to the date of prescription from 2014–2023. The Dispensed Price for Maximum Quantity (DPMQ) was used to estimate the costs of each antiviral and other medicine. The unit cost of medicine=DPMQ/Maximum quantity of packs. The costs were estimated based on duration of the medication use within each financial year. If a medication usage period spanned multiple financial years, the total cost was distributed proportionally based on the number of days in each respective year.

1. Summarise cost of antivirals pre-and post-Hep B PAST

Antiviral Medications	Pre-Hep B PAST	Post-Hep B PAST
Entecavir 0.5mg	$429,714	$658,593
Entecavir 1mg	0	$1,776
Tenofovir disoproxil fumarate 300mg	$102,171	$275,715
**Total**	**$531,885**	**$936,084**

2. Summarise cost of other liver related medications pre-and post-Hep B PAST

Other Liver Related Medications (except antivirals)	Pre-Hep B PAST	Post-Hep B PAST
Furosemide 20mg tablet	$2,085	$2,779
Furosemide 20mg/2mL	$87	$3,413
Furosemide 40mg tablet	$4,861	$5,523
Furosemide 500mg	0	$1,485
Lactulose 3.34g/5mL	$1,684	0
Norfloxacin 400mg	$415	$131
Propranolol 10mg	0	$1,147
Propranolol 40mg	0	$1,069
Rifaximin 550mg	0	$36,458
Spironolactone 100mg	$322	$521
Spironolactone 12.5	$175	$2,859
Spironolactone 25mg	$2,051	$1,929
Sulfamethoxazole 200mg/5mL + Trimethoprim 40mg/5mL	$70	$68
Sulfamethoxazole 400mg + Trimethoprim 80mg	0	$97
Sulfamethoxazole 800mg + Trimethoprim 160mg	$4,744	$8,938
**Total**	**$16,494**	**$66,419**

Table S7. Sensitivity analyses of total healthcare costs for outreach and primary care services pre-and post-Hep B PAST

Scenario	Financial Year	Pre-Hep B PAST						Post-Hep B PAST					
		Number of Individual (Contact)	Total Cost (Pre)	Mean cost/ Contact/ year (95% CI)	Median cost/ Contact/year (IQR)	Mean cost/ Individual/ year (95% CI)	Median cost/ Individual/ year (IQR)	Number of Individual (Contact)	Total Cost (Post)	Mean cost/ Contact/year (95% CI)	Median cost/ Contact/year (IQR)	Mean cost/ Individual/ year (95% CI)	Median cost/ Individual/ year (IQR)
Outreach cost of A$400	2014/15	436 (975)	576,966	592 (479; 705)	317 (317; 317)	1,323 (916; 1,731)	574 (317; 951)	0	0	0	0	0	0
Outreach cost of A$400	2015/16	519 (1,163)	870,034	748 (595; 902)	322 (322; 322)	1,676 (1,134; 2,219)	644 (322; 1,164)	0	0	0	0	0	0
Outreach cost of A$400	2016/17	458 (1,128)	913,802	810 (657; 964)	325 (325; 392)	1,995 (1,515; 2,476)	650 (325; 2,031)	28 (37)	63,984	1,729 (-1,084; 4,543)	325 (325; 325)	2,285 (-1,563; 6,134)	325 (325; 325)
Outreach cost of A$400	2017/18	405 (835)	576,712	691 (570; 812)	331 (331; 331)	1,424 (1,053; 1,795)	331 (331; 928)	106 (338)	161,481	478 (397; 559)	331 (331; 331)	1,523 (961; 2,086)	662 (402; 1,586)
Outreach cost of A$400	2018/19	178 (329)	297,890	905 (480; 1,331)	338 (338; 338)	1,674 (825; 2,523)	338 (338; 993)	292 (740)	479,492	648 (426; 870)	338 (338; 338)	1,642 (935; 2,349)	676 (338; 1,014)
Outreach cost of A$400	2019/20	110 (188)	233,358	1,241 (330; 2,152)	343 (343; 343)	2,121 (-20; 4,263)	343 (343; 577)	566 (1,665)	935,747	562 (460; 664)	343 (256; 343)	1,653 (1,224; 2,083)	686 (343; 1,196)
Outreach cost of A$400	2020/21	0	0	0	0	0	0	632 (2,024)	1,459,031	721 (635; 807)	342 (342; 342)	2,309 (1,606; 3,011)	684 (342; 1,368)
Outreach cost of A$400	2021/22	0	0	0	0	0	0	666 (1,849)	1,331,444	720 (598; 842)	355 (355; 355)	1,999 (1,480; 2,519)	710 (355; 1,166)
Outreach cost of A$400	2022/23	0	0	0	0	0	0	594 (1,595)	1,040,086	652 (559; 745)	377 (242; 377)	1,751 (1,268; 2,234)	633 (377; 1,124)
**Outreach cost of A$400 (Total)**		**859 (4,618)**	**3,468,760**	**751 (672; 830)**	**325 (317; 338)**	**1,647 (1,406; 1,888)**	**597 (325; 1,113)**	**926 (8,248)**	**5,471,265**	**663 (613; 713)**	**343 (331; 377)**	**1,897 (1,649; 2,145)**	**684 (355; 1,162)**
Outreach cost of A$500	2014/15	436 (975)	625,946	642 (530; 754)	396 (396; 396)	1,436 (1,028; 1,843)	653 (396; 1,188)	0	0	0	0	0	0
Outreach cost of A$500	2015/16	519 (1,163)	924,194	795 (641; 948)	402 (402; 402)	1,781 (1,238; 2,323)	804 (402; 1,323)	0	0	0	0	0	0
Outreach cost of A$500	2016/17	458 (1,128)	964,427	855 (702; 1,008)	406 (406; 406)	2,106 (1,625; 2,586)	812 (406; 2,197)	28 (37)	66,252	1,791 (-1,019; 4,601)	406 (406; 406)	2,366 (-1,488; 6,221)	406 (406; 406)
Outreach cost of A$500	2017/18	405 (835)	618,627	741 (621; 861)	414 (414; 414)	1,527 (1,156; 1,899)	414 (414; 1,063)	106 (338)	179,990	533 (453; 612)	414 (414; 414)	1,698 (1,131; 2,265)	828 (485; 1,813)
Outreach cost of A$500	2018/19	178 (329)	315,825	960 (536; 1,384)	423 (423; 423)	1,774 (926; 2,622)	423 (423; 1,122)	292 (740)	520,547	703 (482; 925)	423 (423; 423)	1,783 (1,075; 2,490)	846 (423; 1,269)
Outreach cost of A$500	2019/20	110 (188)	244,452	1,300 (390; 2,210)	429 (429; 429)	2,222 (80; 4,364)	429 (429; 642)	566 (1,665)	1,016,845	611 (509; 712)	429 (256; 429)	1,797 (1,365; 2,228)	858 (429; 1,346)
Outreach cost of A$500	2020/21	0	0	0	0	0	0	632 (2,024)	1,558,447	770 (684; 856)	428 (428; 428)	2,466 (1,762; 3,169)	856 (428; 1,508)
Outreach cost of A$500	2021/22	0	0	0	0	0	0	666 (1,849)	1,424,271	770 (649; 892)	444 (444; 444)	2,139 (1,619; 2,658)	888 (444; 1,344)
Outreach cost of A$500	2022/23	0	0	0	0	0	0	594 (1,595)	1,124,351	705 (613; 797)	472 (242; 472)	1,893 (1,410; 2,376)	728 (472; 1,291)

Scenario	Financial Year	Pre-Hep B PAST						Post-Hep B PAST					
		**Number of Individual (Contact)**	**Total Cost (Pre)**	**Mean cost/ Contact/ year (95% CI)**	**Median cost/ Contact/year (IQR)**	**Mean cost/ Individual/ year (95% CI)**	**Median cost/ Individual/ year (IQR)**	**Number of Individual (Contact)**	**Total Cost (Post)**	**Mean cost/ Contact/year (95% CI)**	**Median cost/ Contact/year (IQR)**	**Mean cost/ Individual/ year (95% CI)**	**Median cost/ Individual/ year (IQR)**
**Outreach cost of A$500 (Total)**		**859 (4,618)**	**3,693,469**	**800 (721; 878)**	**406 (396; 423)**	**1,754 (1,513; 1,995)**	**680 (406; 1,255)**	**926 (8,248)**	**5,890,703**	**714 (664; 764)**	**429 (375; 472)**	**2,043 (1,794; 2,291)**	**856 (444; 1,411)**
Primary care sensitivity	2014/15	436 (975)	603,841	619 (507; 732)	356 (356; 356)	1,385 (977; 1,792)	637 (356; 1,068)	0	0	0	0	0	0
Primary care sensitivity	2015/16	519 (1,163)	899,251	773 (620; 927)	362 (362; 362)	1,733 (1,190; 2,275)	724 (362; 1,273)	0	0	0	0	0	0
Primary care sensitivity	2016/17	458 (1,128)	940,090	833 (680; 987)	366 (366; 392)	2,053 (1,572; 2,533)	732 (366; 2,133)	28 (37)	65,148	1,761 (-1,051; 4,572)	366 (366; 366)	2,327 *(-1,525; 6,178)*	366 *(366; 366)*
Primary care sensitivity	2017/18	405 (835)	597,967	716 (596; 837)	373 (373; 373)	1,476 (1,105; 1,848)	373 (373; 1,012)	106 (338)	170,901	506 (425; 586)	373 (373; 373)	1,612 (1,047; 2,177)	746 (446; 1,712)
Primary care sensitivity	2018/19	178 (329)	306,953	933 (508; 1,358)	380 (380; 380)	1,724 (876; 2,573)	380 (380; 1,102)	292 (740)	500,474	676 (455; 898)	380 (380; 380)	1,714 (1,007; 2,421)	760 (380; 1,140)
Primary care sensitivity	2019/20	110 (188)	238,905	1,271 (360; 2,181)	386 (386; 386)	2,172 (30; 4,314)	386 (386; 610)	566 (1,665)	977,164	587 (485; 689	386 (256; 386)	1,726 (1,296; 2,157)	772 (386; 1,283)
Primary care sensitivity	2020/21	0	0	0	0	0	0	632 (2,024)	1,516,432	749 (663; 835)	385 (385; 385)	2,399 (1,696; 3,103)	770 (385; 1,480)
Primary care sensitivity	2021/22	0	0	0	0	0	0	666 (1,849)	1,378,217	745 (624; 867)	400 (400; 400)	2,069 (1,550; 2,589)	800 (400; 1,252)
Primary care sensitivity	2022/23	0	0	0	0	0	0	594 (1,595)	1,077,990	676 (583; 768)	424 (221; 424)	1,815 (1,332; 2,298)	666 (424; 1,248)
**Primary care sensitivity (Total)**		**859 (4,618)**	**3,587,006**	**777 (698; 855)**	**366 (356; 380)**	**1,703 (1,462; 1,944)**	**639 (366; 1,179)**	**926 (8,243)**	**5,686,326**	**689 (640; 739)**	**386 (366; 424)**	**1,972 (1,723; 2,220)**	**770 (400; 1,273)**

Part 3. References

1. Independent Health and Aged Care Pricing Authority. National Efficient Cost Determination 2023–24. Canberra, Australia: Independent Health and Aged Care Pricing Authority; 2023. Available at: https://www.ihacpa.gov.au/resources/national-efficient-cost-determination-2023-24. Viewed August 2026.
2. Independent Health and Aged Care Pricing Authority. NWAU calculators: Independent Health and Aged Care Pricing Authority; 2023 https://www.ihacpa.gov.au/health-care/pricing/nwau-calculators. Viewed June 2024.
3. Independent Hospital Pricing Authority. Australian Refined Diagnosis Related Groups Version 11.0 Final Report. Sydney: Independent Hospital Pricing Authority; 2022. Available at: https://www.ihacpa.gov.au/sites/default/files/2022-08/ar-drg_v11.0_final_report.pdf. Viewed June 2025.
4. Australian Bureau of Statistics. Consumer Price Index, Australia: Australian Bureau of Statistics; 2023 https://www.abs.gov.au/statistics/economy/price-indexes-and-inflation/consumer-price-index-australia/sep-quarter-2023. Viewed July 2024.
5. Independent Health and Aged Care Pricing Authority. Australian Emergency Care Classification Version 1.1 Final Report. Sydney: Independent Health and Aged Care Pricing Authority; 2024. Available at: https://www.ihacpa.gov.au/sites/default/files/2024-08/australian_emergency_care_classification_version_1.1_final_report.pdf. Viewed June 2025.
6. Independent Health and Aged Care Pricing Authority. Tier 2 Non-Admitted Services Definitions Manual 2023–24. Sydney; 2023. Available at: https://www.ihacpa.gov.au/sites/default/files/2023-03/tier_2_non-admitted_services_definition_manual_2023-24_0.pdf. Viewed March 2024.
7. Zhao Y, Hanssens P, Byron P and Guthridge S. Cost Estimates of Primary Health Care Activities for Remote Aboriginal Communities in the Northern Territory. Darwin: Department of Health and Community Services, Northern Territory; 2006. Available at: https://digitallibrary.health.nt.gov.au/entities/publication/ececc474-a00c-4dd1-bb08-4ad89c84d08e. Viewed June 2025.
8. Zhao Y, Russell DJ, Guthridge S, et al. Costs and effects of higher turnover of nurses and Aboriginal health practitioners and higher use of short-term nurses in remote Australian primary care services: an observational cohort study. *BMJ Open*. 2019;9(2).e023906.
9. Department of Health and Aged Care. PBS Publications Archive. Canberra, ACT: Department of Health and Aged Care; 2024. Available at: https://www.pbs.gov.au/info/publication/schedule/archive. Viewed December 2024.
